# Orai3-Mediates Cisplatin-Resistance in Non-Small Cell Lung Cancer Cells by Enriching Cancer Stem Cell Population through PI3K/AKT Pathway

**DOI:** 10.3390/cancers13102314

**Published:** 2021-05-12

**Authors:** Hiba Abou Daya, Sana Kouba, Hakim Ouled-Haddou, Nazim Benzerdjeb, Marie-Sophie Telliez, Charles Dayen, Henri Sevestre, Loïc Garçon, Frédéric Hague, Halima Ouadid-Ahidouch

**Affiliations:** 1 Laboratoire de Physiologie Cellulaire et Moléculaire, Équipe d’Accueil (EA-4667), Université de Picardie Jules Verne, F-80000 Amiens, France; hiba.abou.daya@etud.u-picardie.fr (H.A.D.); sana.kouba@u-picardie.fr (S.K.); marie-sophie.telliez@u-picardie.fr (M.-S.T.); 2 HEMATIM—UR 4666 Centre Universitaire de Recherche en Santé, Université de Picardie Jules Verne, F-80000 Amiens, France; hakim.ouled-haddou@u-picardie.fr (H.O.-H.); garcon.loic@chu-amiens.fr (L.G.); 3 Service d’Anatomie et Cytologie Pathologiques, Centre Hospitalier Universitaire, Lyon-Sud, F-69002 Lyon, France; nazim.benzerdjeb@chu-lyon.fr; 4 Centre Pour Innovation en Cancérologie de Lyon (CICLY), Équipe d’Accueil (EA-3738), Université Lyon 1, F-69002 Lyon, France; 5 Service de Pneumologie, Centre Hospitalier Saint-Quentin-Picardie, F-02321 Saint-Quentin, France; dayen.charles@live.fr; 6 Service d’Anatomie et Cytologie Pathologiques, Centre Hospitalier Universitaire Amiens-Picardie, F-80000 Amiens, France; sevestre.henri@chu-amiens.fr

**Keywords:** Orai3, store-operated calcium entry, cancer stem cell markers, non-small cell lung carcinoma, Cisplatin, chemoresistance

## Abstract

**Simple Summary:**

Lung cancer is recognized for having a very poor prognosis with an overall survival rate of 5-years not exceeding 15%. Platinum-doublet therapy is the most current chemotherapeutic treatment used to treat lung tumors. However, resistance to such drugs evolves rapidly in patients with non-small cell lung cancer (NSCLC) and is one of the major reasons behind therapy failure. Tumor recurrence due to chemoresistance is mainly attributed to the presence of cancer stem cells (CSCs) subpopulations. Thus, the identification of resistance actors and markers is necessary. The Orai3 channel has been recently identified as a predictive marker of metastasis and survival in resectable NSCLC tumors. Our results show, for the first time, that the Orai3 channel is able to induce chemoresistance by enriching CSCs population. Our findings present Orai3 as a promising predictive biomarker which could help with selecting chemotherapeutic drugs.

**Abstract:**

The development of the resistance to platinum salts is a major obstacle in the treatment of non-small cell lung cancer (NSCLC). Among the reasons underlying this resistance is the enrichment of cancer stem cells (CSCs) populations. Several studies have reported the involvement of calcium channels in chemoresistance. The Orai3 channel is overexpressed and constitutes a predictive marker of metastasis in NSCLC tumors. Here, we investigated its role in CSCs populations induced by Cisplatin (CDDP) in two NSCLC cell lines. We found that CDDP treatment increased Orai3 expression, but not Orai1 or STIM1 expression, as well as an enhancement of CSCs markers. Moreover, Orai3 silencing or the reduction of extracellular calcium concentration sensitized the cells to CDDP and led to a reduction in the expression of Nanog and SOX-2. Orai3 contributed to SOCE (Store-operated Calcium entry) in both CDDP-treated and CD133^+^ subpopulation cells that overexpress Nanog and SOX-2. Interestingly, the ectopic overexpression of Orai3, in the two NSCLC cell lines, lead to an increase of SOCE and expression of CSCs markers. Furthermore, CD133^+^ cells were unable to overexpress neither Nanog nor SOX-2 when incubated with PI3K inhibitor. Finally, Orai3 silencing reduced Akt phosphorylation. Our work reveals a link between Orai3, CSCs and resistance to CDDP in NSCLC cells.

## 1. Introduction

Lung cancer continues to be the deadliest cancer worldwide and the most frequently diagnosed cancer type in both sexes [1]. Among the different histological types of lung cancer, non-small cell lung cancer (NSCLC) comprises the majority of cases (about 85%) including adenocarcinomas, squamous cell carcinomas, and large cell carcinomas [2]. The severity of this type relies notably on the late diagnosis which hinders the surgical operation and restricts the treatments to radiotherapy and chemotherapy. Since several decades, CDDP-based chemotherapy regimens proved to increase the average survival in patients with NSCLC [3]. Recently documented, the combination of platinum salt with third generation drugs (such as pemetrexed, paclitaxel and docetaxel) is still considered as the standard first-line treatment [4], especially for NSCLC tumors lacking a driver mutation or tumors whose immunohistochemistry does not reveal a significant expression of PDL-1 protein. Recently, in 2019, clear evidence about the reliance of the ongoing remedies on platinum-based combos in the world was published [5]. Despite the fact that the treatment with such compounds has proved its efficiency, it is often accused for its unfavorable side effect which is chemoresistance, one of the main reasons of treatment failure. Statistics reveal that up to 43% of NSCLC patients endure tumor recurrence due to chemoresistance [6].

Cancer stem cells (CSCs), described as a small subset of tumor cells with stem cell characteristics, are considered to be one of the major causes of tumor rebound after chemotherapy. It has been reported that treatment with CDDP increases the expression of CSCs markers in NSCLC [7]. Different markers were identified as CSCs markers, but no common universal marker has been approved. CD133 is a cell-surface glycoprotein, known also as prominin-1, that consists of five transmembrane domains and two large glycosylated extracellular loops whose exact role has not been established yet. However, many tumors of variable origins express the membrane CD133 antigen, which is also expressed by normal stem cells of different lineages [8]. It has been used to detect and isolate CSCs from many cancer types including lung cancers [9]. Furthermore, in vitro CDDP treatment of lung cancer cells enhanced the CD133^+^ cell population which was found to be highly tumorigenic, possessing stem-like aspects, and highly resistant to in vivo CDDP exposure of lung tumor xenografts obtained from primary tumors [9].

CSCs can be differentiated from cancer cells by a wide range of identified markers, some of which act as transcription factors. In particular, CSCs which were isolated from primary tumors of NSCLC using CD133, expressed higher levels of Nanog and SOX-2 transcription factors [10]. These two transcription factors are not only required for maintaining the self-renewal in stem cells, but also for the signaling pathways regulating cell differentiation and self-renewal processes [11,12,13].

As a ubiquitous second messenger, calcium is able to control critical cellular processes such as proliferation and apoptosis [14]. Thus, any imbalance in the intracellular calcium concentrations will definitely perturb these two fundamental processes which would probably lead to cancer or worsen the cancer state. Intracellular calcium alterations can be caused by the aberrant expression of calcium channels which is one of the significant hallmarks of cancer [15] and their implication in resistance to chemotherapy was reported by different research groups [16]. Nevertheless, few studies revealed a relationship between calcium signaling or calcium channels and CSCs. Recently, a study has shown that the L- and T-type voltage gated calcium channel (VGCC) genes are overexpressed in ovarian CSCs and the downregulation of those genes impaired the stem cell like properties [17]. Moreover, the VGCC α2δ1 subunit (an auxiliary subunit of VGCC demonstrated as a controller of channel localization and trafficking) was identified as a marker of stemness in gastric CSCs which is involved chemoresistance and in maintaining the CSCs characteristics [18]. The VGCC α2δ1 was also found to be involved in tumorigenesis and resistance to chemotherapy (etoposide and CDDP) in SCLC. In particular, α2δ1^+^ cells were enriched after the treatment, overexpressing CSCs and chemoresistance genes [19]. Additionally, it has been established in oral/oropharyngeal squamous cell carcinoma (OSCC) that Orai1 channel is overexpressed in CSCs [20]. Moreover, Orai1 was shown to be involved in enhancing CSCs where the overexpression of Orai1 in normal nontumorigenic oral epithelial cells stimulated the formation of cell populations with stem cell characteristics expressing upregulated stem cell markers and transcription factors and showed self-renewal abilities. This effect was assured upon the silencing of Orai1 which resulted in the loss of CSCs properties.

The Orai3 channel was found to be overexpressed in tumor lung tissues when compared to nontumoral ones and its expression was correlated with higher tumor grades [21]. In the same study, this channel was shown to be able to regulate cell proliferation through AKT pathway in two adenocarcinoma cell lines [21]. Furthermore, the Orai3 channel was recently recognized as a prognostic marker of metastasis and survival in lung adenocarcinoma [22]. Moreover, the Orai3 channel was implicated in resistance to chemotherapeutic drugs (including CDDP) in breast cancer cells [23]. To the best of our knowledge, neither of the Orai isoforms were previously investigated in the context of CDDP-resistance and CSCs. Until now, only two contradictory studies concerning the involvement of SOCE in CDDP-resistance have been reported in NSCLC, A549 cell line. In 2013, Li et al. showed that SOCE inhibition, using pharmacological inhibitors or STIM1 silencing (siSTIM1), enhanced Cisplatin-induced apoptosis [24]. On the contrary, later in 2019, Gualdani et al. showed that SOCE inhibition using siTRPC1 or siSTIM1 was able to reduce CDDP-cytotoxicity [25]. In the present study, we aimed to investigate the functional role of Orai3 in resistance to CDDP through the regulation of cancer stem cells markers expression. We showed, for the first time, that the Orai3 channel is expressed, functional and favors the survival of CDDP-treated A549 and H23 cells unlike Orai1. In addition, CDDP-treatment induced a positive feedback loop which leads to the enhancement of Orai3 expression, as well as the stem cell markers Nanog and SOX-2 and SOCE. Moreover, ectopic overexpression of Orai3 in H23 and A549 cell lines induced an increase of CD133^+^ cell population, SOX-2 and Nanog expression. Notably, Orai3, SOX-2 and Nanog were highly expressed in the CD133^+^ cell population and silencing of Orai3 decreased Nanog and SOX-2 expression and sensitized the cells to CDDP. Our data also reveal the involvement of PI3K/AKT pathway, via Orai3, in the increase of CSCs markers expression by CDDP.

## 2. Results

### 2.1. Orai3 and Orai1 Expressions in Primary Lung Adenocarcinoma at Advanced Stage before and after Chemotherapy Treatment

**Figure 1:**
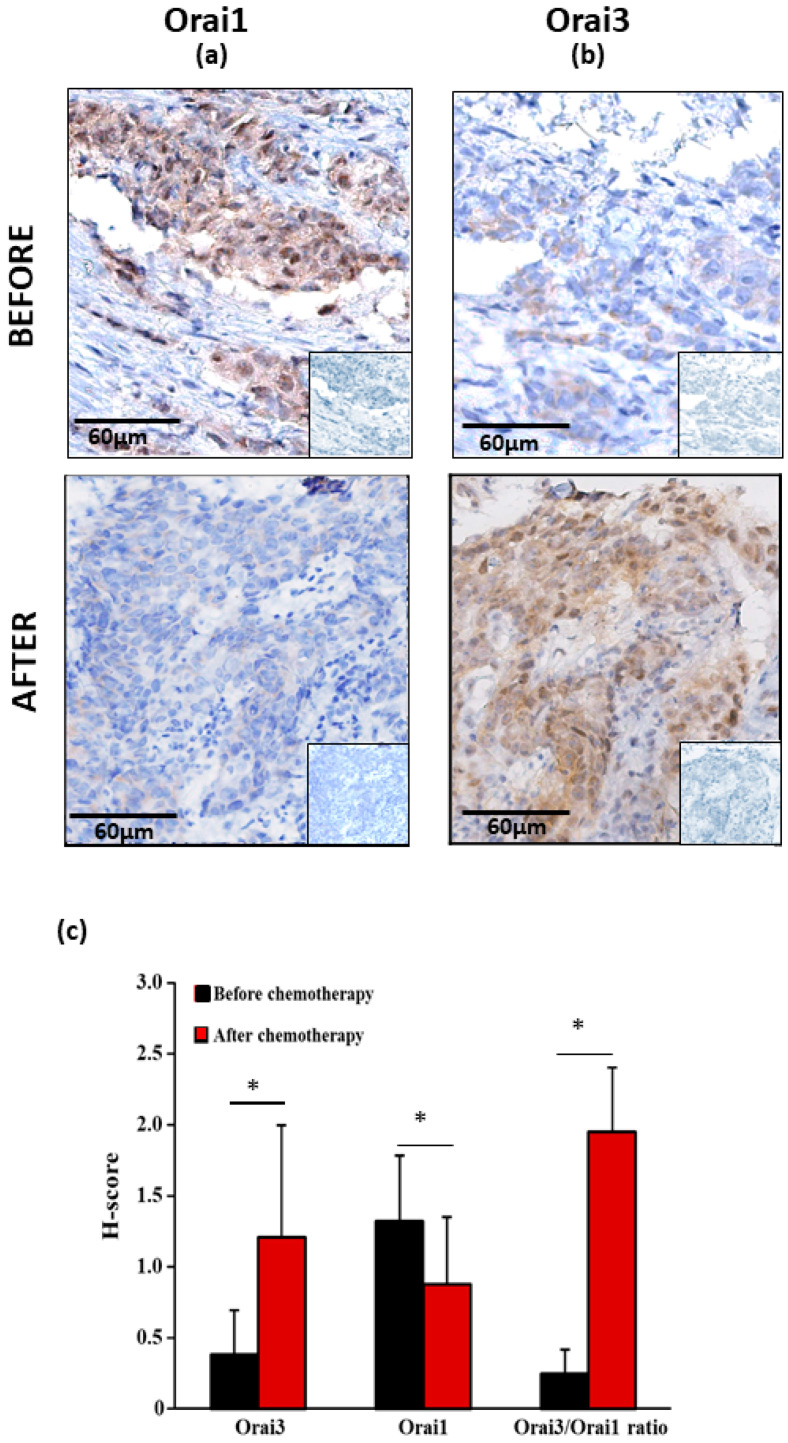
Immunohistochemical staining of Orai1 and Orai3 of bronchial biopsies before and after chemotherapy. (**a**) Representative examples of Orai1 and Orai3 (**b**) expressions of original magnification: × 200. Inserts show negative controls obtained by omitting the primary antibodies. All pictures show a low magnification image of negative controls in the lower right corner. Analysis of H-score of Orai1 and Orai3 of bronchial biopsies before and after chemotherapy (**c**). Values are presented as mean of results obtained from 15 patients ± SEM, * *p* < 0.05, Mann–Whitney U test.

Figure 1 shows the mean of the staining score of both Orai1 and Orai3 in a cohort of 15 patients with a primary lung adenocarcinoma at advanced stage (Figure 1a,b). Before chemotherapy, the staining score of Orai1 was higher than the one of Orai3 (Figure 1c, 1.33 ± 0.46 vs. 0.39 ± 0.31; *p* < 0.05, Mann–Whitney U test). After chemotherapy, the staining score of Orai3 increased by 310.2% (Figure 1c, 0.39 ± 0.31 vs. 1.21 ± 0.79; *p* < 0.05, Mann–Whitney U test) and the one of Orai1 decreased by 33.8% (Figure 1c, 1.33 ± 0.46 vs. 0.88 ± 0.48 *p* < 0.05, Mann–Whitney U test). The ratio Orai3:Orai1 before and after chemotherapy shifted from 0.25 to 1.95 (Figure 1c, 0.25 ± 0.17 vs. 1.95 ± 0.45; *p* < 0.05, Mann–Whitney U test). The H-score of Orai1 and Orai3 was not associated with complete response or partial response/progressive disease of RECIST1.1. The Response Evaluation Criteria in Solid Tumors (RECIST) is a standardized methodology using validated criteria to evaluate the response of a tumor to treatment in solid tumors [26].

### 2.2. Orai3 Silencing, but Not Orai1, Sensitized Lung Cancer Cells to CDDP-Induced Apoptosis

**Figure 2:**
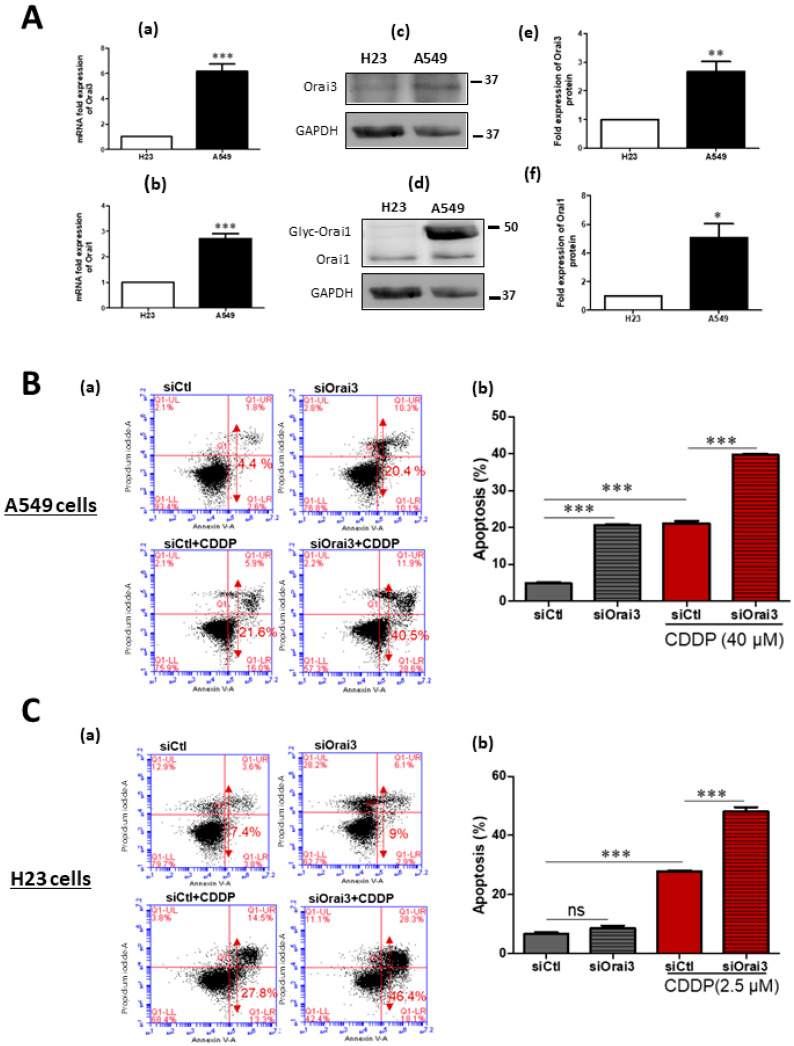
Orai3 silencing increases the sensitivity to CDDP treatment in A549 and H23 cells. (**A**) Orai3 and Orai1 expression in A549 compared to H23 cell line. Expression at the mRNA level of Orai3 (**A**-**a**) and Orai1 (**A**-**b**) normalized to GAPDH. Western blot showing protein expression of Orai3 (**A**-**c**) and Orai1 (**A**-**d**) with their respective quantifications (**A**-**e**) and (**A**-**f**). Values presented are the mean of three independent experiments ± SEM. *** *p* < 0.001, ** *p* ˂ 0.01, * *p* ˂ 0.05, Student’s t-test. (**B**,**C**) Assessment of CDDP-induced apoptosis using flow cytometry Annexin V and PI staining in A549 (**B-a**) and H23 (**C-a**) cells. Quantification of the percentage of apoptosis in each condition in A549 (**B-b**) and H23 (**C-b**) cells. Values of three independent experiments are presented as mean ± SEM. *** *p* < 0.001 vs. control, ns: not significant. ANOVA followed by Holm–Sidak post test.

Before studying the potential role of the calcium channel Orai3 in CDDP-induced apoptosis we determined the IC**_50_** (the concentration at which 50% of the effect is obtained) of CDDP after 48 h treatment by measuring the cellular viability by MTT test. A549 cells revealed a high resistance to CDDP with an IC**_50_** of 40 μM, while H23 cells were much more sensitive to Cisplatin with an IC_50_ of 2.5 μM (Figure S1A,B). We then investigated whether CDDP-induced mortality is dependent on extracellular Ca^2+^. The obtained results show that low external calcium leads to a similar percentage increase in mortality of both cell lines, H23 (32%) and A549 (30%) in the presence of CDDP. In H23 cells, the Cisplatin-mortality rate increased from 25.33 ± 0.88% to 33.33 ± 0.88% under normal and low extracellular calcium, respectively (Figure S1C). In A549 cells, low extracellular calcium induced a higher mortality rate of 65.30 ± 1.91% compared to the 50.07 ± 2.82% caused by Cisplatin under physiological calcium concentration (Figure S1D). We also realized that only in the case of A549 cells, a lower extracellular calcium concentration increases the cellular mortality in basal culture conditions where an increase from 2.04 ± 0.095% to 8.53 ± 0.48% was observed. The expression of Orai3 and Orai1 has been previously reported by Ay and colleagues in the two NSCLC cell lines H23 and H460, but not in A549 cells [21]. First, we compared the expression of Orai3 and Orai1 channels in A549 and H23 cells at the transcript and protein level where we took H23 cells as a control since they belong to the same histological subtype as A549 cells which is the adenocarcinoma while H460 cells belong to large cell carcinoma subtype. There was a significant difference in the expression level between the two cell lines. A549 mRNA expression of Orai3 was 6.1 ± 0.59-fold higher than that of H23 (Figure 2A-a). Orai1 mRNA expression was 2.72 ± 0.196-fold higher in A549 than H23 cells (Figure 2A-b). At the protein level, Orai3 and Orai1 protein quantity was also higher in A549 cells than that in H23 cells by 2.66 ± 0.35-fold for Orai3 (Figure 2A-c,e) and 5.1 ± 0.988 for Orai1 (Figure 2A-d,f).

Next, we investigated whether Orai3 has a role in apoptosis resistance after 48 h treatment with CDDP. After transfection, cells were treated during 48 h with CDDP and apoptosis was assessed by flow cytometry. The validation of the efficiency of our siRNA targeting Orai3 gene in both cell lines was performed by qPCR and Western blot (see Figure S2). In H23 cells, Orai3 mRNA and protein levels decreased by 56 ± 3.1% and 43 ± 3.9%, respectively, in cells transfected with siOrai3 compared to cells transfected with siCtl (Figure S2A,B). In A549 cells transfected with siOrai3, Orai3 transcripts and protein decreased by 75 ± 2.8% and 67 ± 5.4%, respectively (Figure S2C,D), without affecting the expression of Orai1 or STIM1 (Figure S2E,F). As observed in the Figure 2B-a,b, after Orai3 silencing and during the incubation with Cisplatin, A549 cell apoptosis was 21.13 ± 0.62% in siCtl compared to 39.63 ± 0.44% in siOrai3 condition (Figure 2B-a,b, *p* < 0.001). Similarly, Orai3 silencing was able to increase the Cisplatin-induced apoptosis in H23 cells from 27.87 ± 0.376% in siCtl to 48.17 ± 1.40% in siOrai3 conditions (Figure 2C-a,b, *p* < 0.001). The obtained data suggest the involvement of Orai3 in resistance to Cisplatin in NSCLC cells. Furthermore, we checked whether Orai1 was involved in CDDP-resistance. Silencing of Orai1 failed to affect the cells’ sensitivity to CDDP in both cell lines (Figure S1E,F).

### 2.3. CDDP-Treatment Increased Orai3 Expression

**Figure 3:**
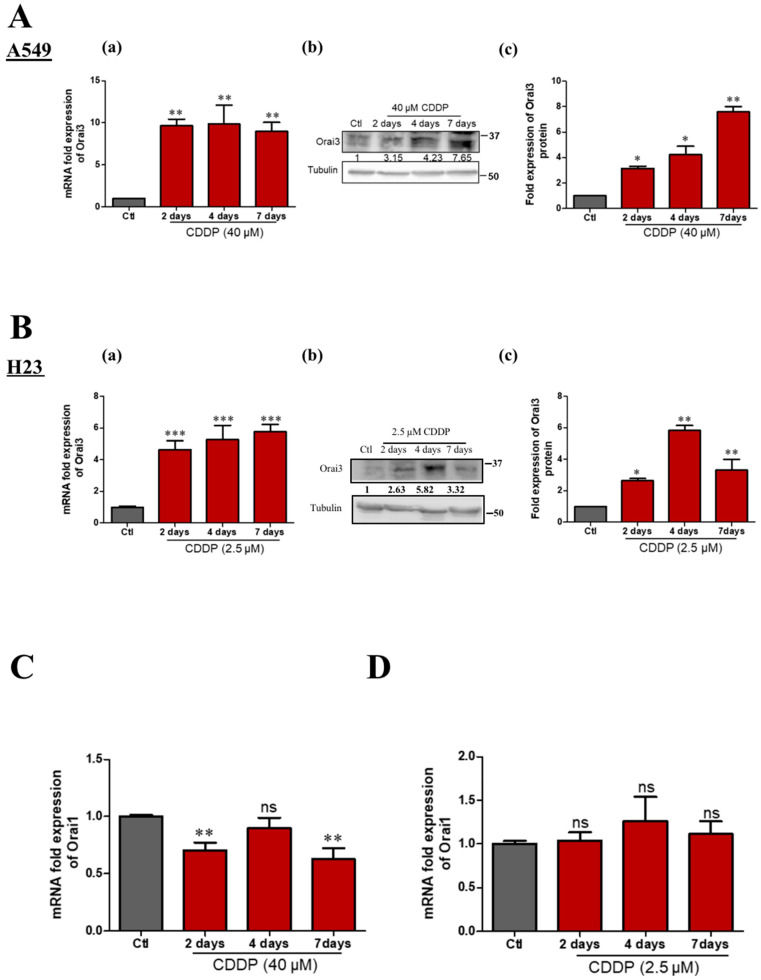
CDDP treatment increases the expression of Orai3 in both NSCLC cell lines. Orai3 gene expression following CDDP treatment in A549 cells (**A-a**). Western blot representing Orai3 protein expression (**A**-**b**) with its respective quantification normalized to Tubulin (**A**-**c**), ANOVA followed by Dunnett post-test ** *p* <0.01, * *p* < 0.05 of at least three independent experiments. Orai3 expression at mRNA level under CDDP treatment in H23 cells (**B-a**), ANOVA followed by Dunnett post-test *** *p* < 0.001 of three independent experiments. Western blot representing Orai3 protein expression (**B**-**b**) with its respective quantification normalized to Tubulin (**B**-**c**), ANOVA followed by Dunnett post-test ** *p* < 0.01, * *p* < 0.05, *N* = 3. (**C,D**) Orai1 mRNA quantification in A549 (**C**) and H23 (**D**) cells treated by CDDP. ANOVA followed by Dunnett post-test ** *p* < 0.01, *N* = 4.

The variation in the expression of Orai1 and Orai3 upon the treatment with chemotherapeutic drugs has been reported in several cancer types. Schmidt and colleagues found that ovarian cancer cells that are resistant to CDDP express higher transcript and protein levels of Orai1, compared to CDDP-sensitive ovarian cancer cells [27]. In breast cancer, bioinformatics analyses revealed that Orai3 mRNA expression was higher in tumors from patients with poor response to therapy than those from patients whose response was good or complete [23]. In contrast, in leukemia, high levels of Orai3 were detected in Tipifarnib-sensitive myeloid cells and resistant cells expressed lower Orai3 levels [28]. We thus examined whether an in vitro treatment with Cisplatin could affect the expression of Orai3, similarly to clinical observations. We found that both Orai3 transcript and protein levels increase significantly during Cisplatin treatment over several days in both cell lines but not those of Orai1. Orai3 transcripts increased by 9.6 ± 0.80, 9.8 ± 2.29 and 8.75 ± 1.11-fold more than the control after 2, 4 and 7 days treatment, respectively, in A549 cells (Figure 3A-a) and at the protein level, a respective fold increase of 3.15 ± 0.18, 4.23 ± 0.66 and 7.6 ± 0.42 was detected (Figure 3A-b,c). A similar trend was observed in H23 cells where the 2, 4 and 7 days Cisplatin treatment increased Orai3 expression, respectively, by 4.65 ± 0.55, 5.27 ± 0.77 and 5.76 ± 0.46-fold at the mRNA level (Figure 3B-a) and 2.63 ± 0.15, 5.82 ± 0.32 and 3.32 ± 0.68-fold at the protein level (Figure 3B-b,c). A continuous and progressive increase of Orai3 protein was remarkable in A549 cells while treatment prolongation until 7 days resulted in a slight drop in Orai3 protein expression in H23, but it was still significantly higher than the control. Moreover, in A549 cells, CDDP induced a slight decrease of the Orai1 transcripts (29 ± 5.4%, and 37.13 ± 7.5% after 2 and 7 days, respectively, but not at 96 h (Figure 3C, *p* < 0.01) or 72 h treatment. However, in H23 cells CDDP failed to affect Orai1 expression (Figure 3D). In both cell lines, the expression of STIM1 and STIM2 is not affected by 48 h CDDP treatment (Figure S1G,H).

### 2.4. Cisplatin by Overexpressing Orai3 Favors Calcium Entry in Both Cell Lines

**Figure 4:**
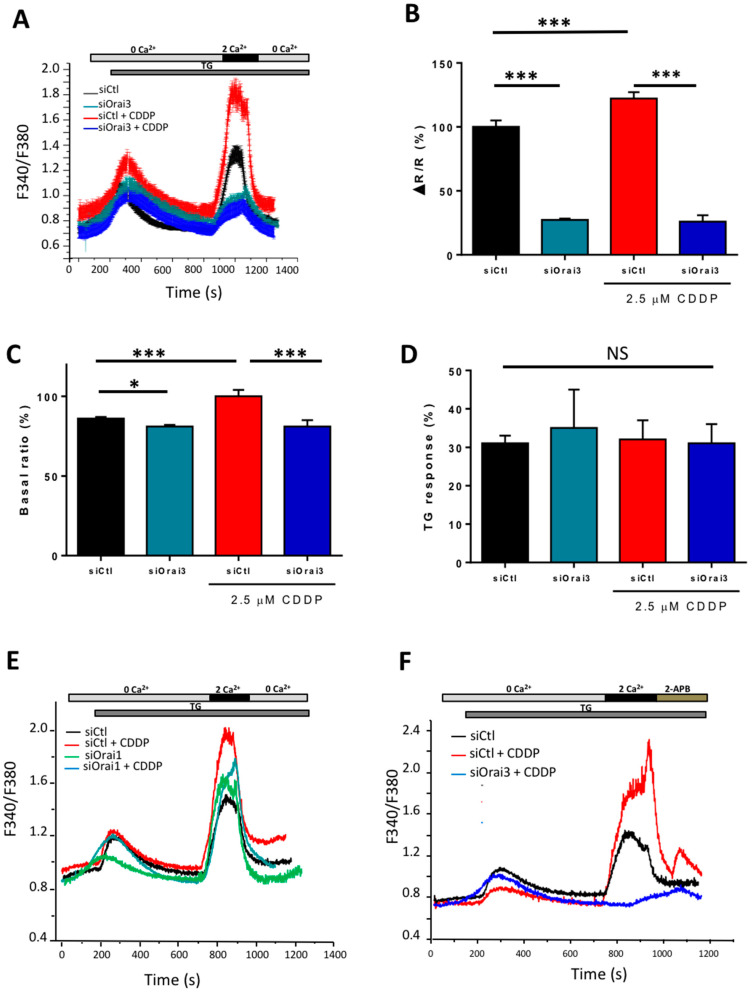
Functional expression of Orai3 and Orai1 in H23 cell line. Traces representing the SOCE measured with the ratio F340/F380 using calcium imaging technique in H23 cells after Orai1 or Orai3 silencing in the presence of CDDP (**A**,**E**,**F**). Cells were exposed to 1 µM TG in the absence of extracellular Ca^2+^ which depletes the intracellular Ca^2+^ stores. Extracellular calcium concentration was then brought to 2 mM in order to induce SOCE (2APB was perfused in **F**). Quantification of SOCE (**B**), basal calcium (**C**) and TG-response (**D**). All histograms are represented as the average ± SEM normalized to the control. (**A**–**D**; siCtl: *n* = 150, siOrai3: *n* = 70, siCtl + CDDP: *n* = 60, siOrai3 + CDDP: *n* = 34, *N* = 3), (**E**; siCtl: *n* = 116, siOrai1: *n* = 40, siCtl + CDDP: *n* = 34, siOrai1+ CDDP: *n* = 25, *N* = 3), (**F**, siCtl: *n* = 90, siCtl + CDDP: *n* = 30, siOrai3 + CDDP: *n* = 26, *N* = 3). Student’s *t*-test, * *p* < 0.05, *** *p* < 0.001, N.S. statically not significant, *n*: number of cells, *N*: number of passages.

After demonstrating the upregulation of Orai3 channel in H23 and A549 cells by qPCR and Western blot upon the incubation with Cisplatin, we investigated its functionality under CDDP condition. Calcium imaging experiments were carried out on both cell lines transfected with siCtl and siOrai3 and exposed to 2.5 and 40 µM CDDP for 72 h for H23 and A549 cells, respectively. The SOCE observed after the perfusion of 2 mM extracellular Ca^2+^ in the presence of Thapsigargin in H23 siCtl cells cultured in a medium containing Cisplatin, is superior to that registered in siCtl cells cultured in normal culture medium by 22.65 ± 4.6% (Figure 4A,B). We also investigated if CDDP leads to differences in Ca^2+^-release from intracellular Ca^2+^ stores (response to TG) or in basal calcium concentration (basal ratio). For the TG-response, we subtracted base lines from TG-induced Ca^2+^ peaks in 0 Ca^2+^ for single cells and quantified the averages. CDDP increased the basal ratio by 16% but failed to affect the TG-response (Figure 4C,D). Indeed, we observed a strong variability in the TG-response in CDDP-treated cells and the mean was not statically significant. The downregulation of Orai3 by siRNA similarly reduced SOCE, and basal calcium concentration in cells treated or not by CDDP (Figure 4B,C). The amplitude of the TG-response was not statically affected in siOrai3 cells treated by CDDP but its kinetic appears to be slower (Figure 4D). However, Orai1 silencing failed to affect SOCE in the Ctl conditions but induced a reduction of 16.4 ± 2.07% which remains statically significant under CDDP (Figure 4E). Furthermore, the perfusion of 2-APB (50 µM) in cells treated by CDDP induced a large potentiation of SOCE that is completely abolished in siOrai3 cells (Figure 4F).

The contribution of Orai family in SOCE has not been determined in A549 cells. Only one study showed a reduction of SOCE following an ectopic overexpression of Orai1 [29]. We then performed a calcium imaging experiment on A549 cells transfected with siCtl and siOrai3 or siOrai1 to determine their potential involvement in SOCE. We found that siOrai1 decreased SOCE by 38.19 ± 5.19% (Figure S3A,B, *p* < 0.05). Surprisingly, in contrast to H23 cells where silencing of Orai3 in control condition decreased SOCE, we found an increase of SOCE by 23.61 ± 13.61% in siOrai3 A549 cells (Figure S3A,B, *p* < 0.05). A549 cells treated with CDDP during 72 h showed a higher SOCE entry of 176.67 ± 11.61% (Figure S3D) without any significant effect on basal calcium concentration and the TG-response (Figure S3E,F). Moreover, SOCE was lower by 37 ± 5% in cells lacking Orai3 (Figure S3C,D). Silencing of Orai1 also reduced SOCE in CDDP condition by 45.5 ± 9.01%, but this reduction was less important than that observed in nontreated cells (55.1 ± 6.3%) (Figure S3G). Furthermore, we found a statistically significant reduction in basal calcium concentration in siOrai3 Ctl cells (Figure S3E), but not in the presence of CDDP. Indeed, basal calcium tends to decrease in siOrai3 cells under CDDP, but this result remains statistically insignificant (Figure S3E). We also investigated whether Orai3 can be activated by 2-APB in A549 cells treated and not treated by CDDP in the absence of Thapsigargin-induced store depletion. Figure S3H shows a Fura2 experiment where cells were incubated in medium containing 2 mM Ca^2+^ and stimulated with 2-APB. In this case, 2-APB caused a small but significant increase in intracellular Ca^2+^ in A549 siCtl cells but increased by 87.18% in CDDP conditions (Figure S3I, *p* < 0.0001). Moreover, 2-APB failed to increase calcium concentration in cells lacking Orai3 in the presence or absence of CDDP (Figure S3H,I). These results could mean that Orai3 is involved in the regulation of basal calcium entry in A549 cells. However, any store-depleting effect of 2-APB cannot be ruled out. Taken together, our results showed that the composition of SOC channels is different between H23 and A549 cells, and Cisplatin favors calcium entry in A549 cells by allowing Orai3 to adopt a positive regulatory role, which otherwise assumes a negatively regulating role in nontreated conditions (Figure S3A,B).

### 2.5. Orai3 Overexpression Protects the Cells against Cisplatin-Induced Apoptosis

**Figure 5:**
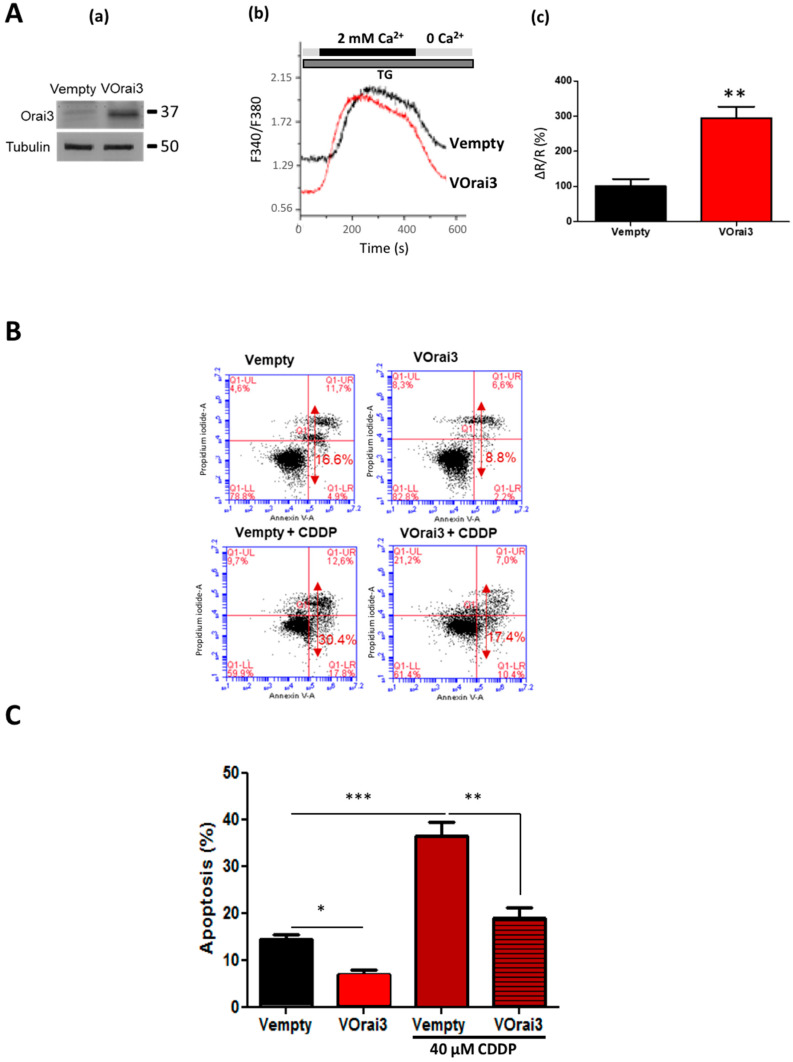
Effect of Orai3 channel overexpression on apoptosis induced by Cisplatin in A549 cells. Transfection validation with VOrai3 via Western blot showing Orai3 protein expression (**A-a**). Effect of Orai3 overexpression on the calcium signal in A549 cells. SOCE was measured after endoplasmic reticulum release by 1µM TG (**A-b**). Quantification of SOCE by measuring the ratio of the peak amplitude over the initial (**A-c**). Student’s *t*-test, ** *p* < 0.01, Vempty: *n* = 53, VOrai3: *n* = 89, *N* = 3. Assessment of CDDP-induced apoptosis using flow cytometry Annexin V and PI staining in A549 cells transfected with Vempty and VOrai3 (**B**). Quantification of the percentage of apoptosis (**C**), ANOVA followed by Holm–Sidak post-test, *** *p* < 0.001 ** *p* < 0.01 * *p* < 0.05, *N* = 3.

As CDDP induced an overexpression of Orai3, and as Orai3 favors the survival of cells treated with CDDP, we investigated the effect of Orai3 overexpression on apoptosis resistance in A549 cells under normal culture conditions and in the presence of CDDP. Cells were transfected with an Orai3 vector (VOrai3) leading to Orai3 overexpression and an empty vector (Vempty) as a control. The overexpression was validated at the protein level with an increase of almost five times more Orai3 protein in VOrai3 cells versus cells transfected with an empty vector (Figure 5A-a). The expression of STIM1 and Orai1 is similar in both VOrai3 and Vempty cells (Figure S2G,H).

Using calcium imaging, VOrai3 was found to increase SOCE by 194.6 ± 39.05% (Figure 5A-b,c, *p* < 0.01). Transfected cells were passed in flow cytometry to detect apoptosis rate after Cisplatin treatment during 48 h. The results revealed that cellular apoptosis caused by Cisplatin was reduced by half when Orai3 was overexpressed decreasing from 36.47 ± 3.05% in Vempty to 18.90 ± 2.18% in VOrai3 cells (Figure 5B,C, *p* < 0.01). Moreover, contrary to siOrai3 which enhanced apoptosis in A549 cells in control conditions (Figure 2B), overexpression of Orai3 in similar conditions reduced the apoptosis from 14.33 ± 1.20% to 6.97 ± 0.93% (Figure 5C, *p* < 0.05). However, the overall apparent decrease in apoptosis in VOrai3 compared to Vempty A549 cells remains similar in both in the absence or presence of CDDP conditions i.e., -2-fold decrease (Figure 5B,C). We also overexpressed Orai3 in H23 cells and performed cell mortality assay under CDDP treatment. CDDP failed to increase cell mortality in VOrai3 cells compared to Vempty cells (Figure S2I).

### 2.6. Orai3 Modulates the Expression of Cancer Stem Cells Markers: Nanog and SOX-2

**Figure 6:**
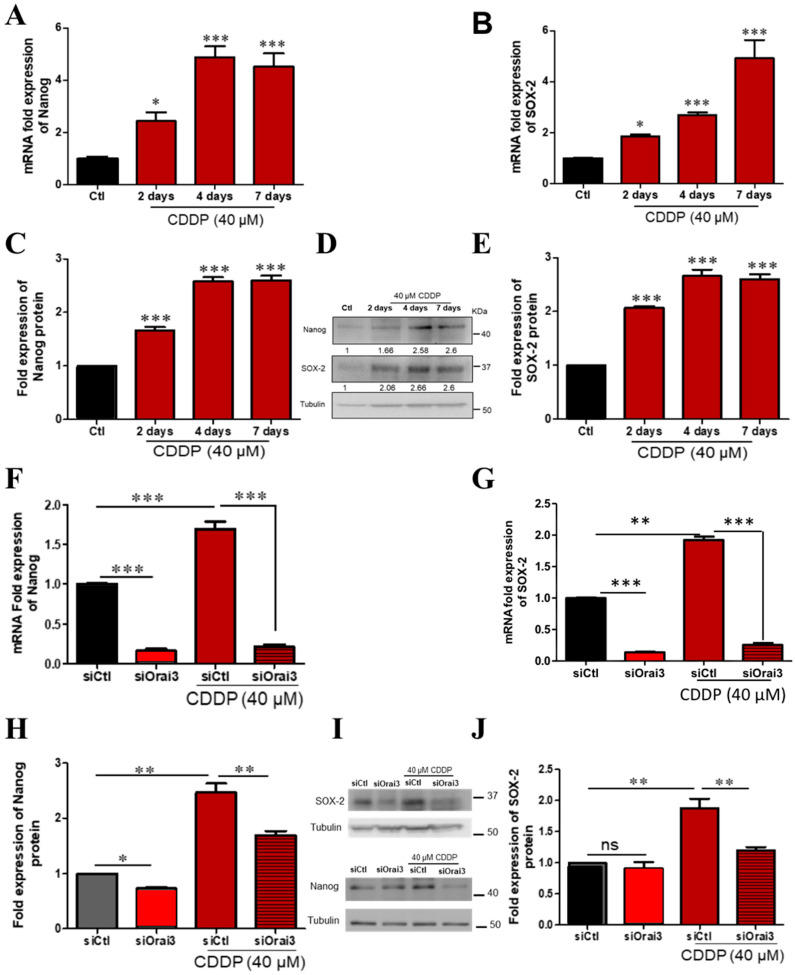
CDDP induces expression of stem cell markers in A549 cells which was prevented upon Orai3 silencing. Relative mRNA expression of Nanog (**A**) and SOX-2 (**B**) after 2–7 days CDDP treatment with respect to GAPDH and relative protein expression (**D**) of Nanog (**C**) and SOX-2 (**E**) normalized to Tubulin, ANOVA followed by Dunnett post-test ** *p* < 0.01, *** *p* < 0.001, *N* = 3. Relative mRNA expression of Nanog (**F**) and SOX-2 (**G**) after Orai3 silencing and 48 h treatment with CDDP and the respective protein expression using Western blot (**I**) representing Nanog (**H**) and SOX-2 (**J**) protein expression normalized to Tubulin, ANOVA followed by Holm–Sidak post-test * *p* < 0.05,** *p* < 0.01, ***p <0.001. ns: not significant, *N* = 3.

We first confirmed the ability of CDDP to increase the expression of SOX-2 and Nanog in both A549 and H23 cells. We followed the expression of Nanog and SOX-2 during the previously described one-week treatment. We found that prolonged CDDP treatment leads to a progressive increase in the mRNA expression of the two markers Nanog and SOX-2. In CDDP-treated A549 cells, Nanog transcript increased by 2.43 ± 0.34-fold 48 h after the treatment and continued to increase to reach 4.51 ± 0.52-fold the control at day 7 (Figure 6A, *p* < 0.05, *p* < 0.001). SOX-2 transcript also increased to become 1.86 ± 0.08-fold the control after 2 days, reaching 4.9 ± 0.72-fold after 7 days (Figure 6B, *p* < 0.05, *p* < 0.001). The observed increase in SOX-2 and Nanog was also detected at the protein level by Western blot. We detected an increase in Nanog protein by 1.66 ± 0.064 and 2.6 ± 0.091-fold the control, 2 and 7 days after CDDP incubation (Figure 6C,D, *p* < 0.001), and SOX-2 protein increased by 2.06 ± 0.032 and 2.6 ± 0.093-fold the control, respectively (Figure 6D,E, *p* < 0.001). In order to confirm this effect, we incubated A549 cells in CDDP at low concentration (5 µM) during 96 h. Our results showed an increase in the expression of Orai3, Nanog and SOX-2 without affecting the expression of STIM1 and Orai1 (Figure S4A). We then checked whether Orai3 is capable of modulating the expression of those markers. We silenced Orai3 and we pursued Nanog and SOX-2 transcript and protein changes 48 h after CDDP treatment. Results showed that Orai3 downregulation decreased the expression of both Nanog and SOX-2 at the transcript and protein levels. The transcript fold expression of Nanog decreased to 0.156 ± 0.03 in control culture medium and to 0.21 ± 0.029 in the presence of CDDP (Figure 6F, *p* < 0.001). Similarly, the transcript level of SOX-2 decreased upon Orai3 silencing in normal medium to 0.17 ± 0.006 and in CDDP-containing medium to 0.29 ± 0.01 (Figure 6G, *p* < 0.001). Orai3 silencing was able to decrease Nanog protein to 1.69 ± 0.074-fold where siCtl cells treated with CDDP recorded 2.47 ± 0.16-fold increase of Nanog protein (Figure 6H,I, *p* < 0.01). The protein expression of SOX-2 which increased 1.88 ± 0.15-fold the control after 48 h exposure to CDDP in siCtl condition, decreased until 1.21 ± 0.047-fold in siOrai3 condition (Figure 6I,J, *p* < 0.01).

With regard to the context of our study, it was critical at this point to figure out if the expression of those markers is affected by extracellular Ca^2+^ concentration. For this, cells were treated during 48 h with CDDP upon the reduction of external Ca^2+^ concentration to 0.5 mM. CDDP failed to increase the expression of Nanog under low calcium concentration where the mRNA expression was 0.84 ± 0.05-fold the control. Similarly, the transcript of SOX-2 was recorded to be 0.69 ± 0.036-fold the control in a low Ca^2+^ medium (Figure S4B,C, *p* < 0.001).

### 2.7. The Increase of Orai3 Expression by CDDP Is Associated with an Increase in Stem Cell Markers

**Figure 7:**
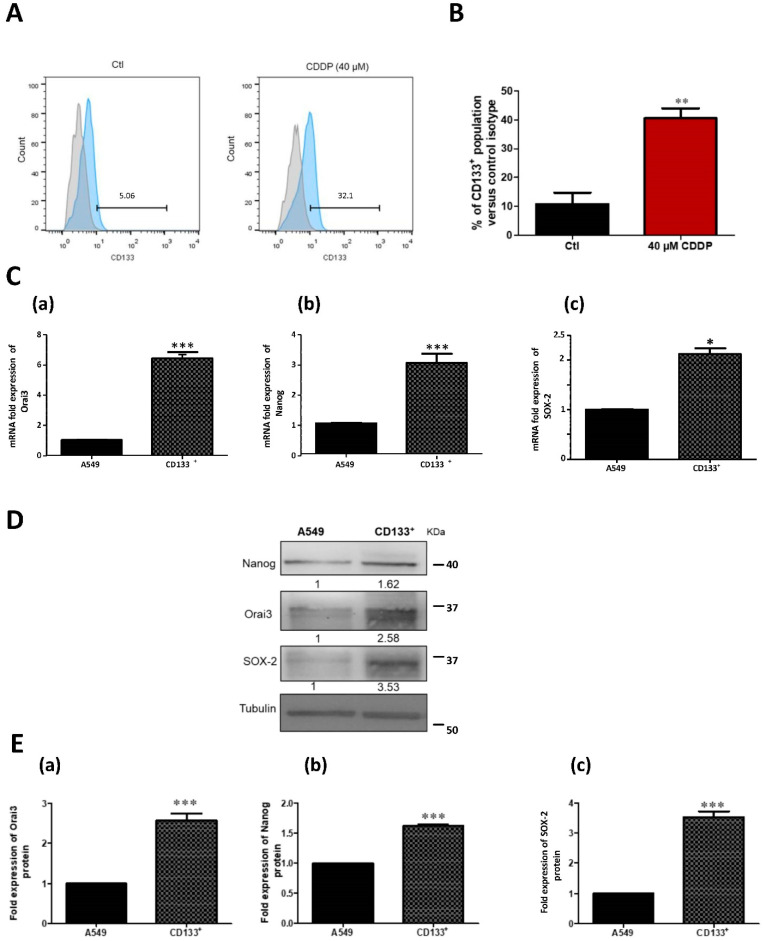
CDDP treatment in A549 cells increased CD133^+^ cell population. Flow cytometry representative histogram showing that 40 µM Cisplatin treatment for 48 h increased CD133 expression with respect to control (**A**) and its respective quantification (**B**). Bars represent percentage values of CD133^+^ population of each condition relative to isotype. Student’s *t*-test, ** *p* < 0.01, *N* = 3. The expression of Orai3 and stemness markers in sorted CDDP-induced CD133^+^ cells *vs*. parental A549 cells. Relative mRNA expression of Orai3 (**C-a**), Nanog (**C-b**) and SOX-2 (**C-c**). Western blot representing Orai3, SOX-2 and Nanog protein expression (**D**) with the respective quantifications (**E-a**, **E-b** and **E-c**). Student’s *t*-test, * *p* < 0.05, *** *p* < 0.001, *N* = 3.

Since CSCs are considered as the major cause of treatment resistance and since our data showed that Orai3 is involved in chemoresistance in NSCLC tissues and cell lines, we thought to study the possible involvement of Orai3 in CSCs enrichment. In order to identify the percentage of stem cell enrichment after the exposure of A549 and H23 cells to CDDP, treated and control cells were labeled by florescent CD133 or CD44 antibody and analyzed by flow cytometry. In A549 cells, the fraction of CD133 expressing cells (CD133^+^) was 10.75 ± 3.97% in control condition and CDDP-treatment extended this population up to 40.50 ± 3.50% confirming a remarkable enrichment in CSCs fraction (Figure 7A,B, *p* < 0.01). After 48 h treatment with CDDP, we sorted CD133^+^ cells and compared them to the parental A549 cells at the level of CSCs markers expression and Orai3 expression. Indeed, CD133^+^ cells expressed higher levels of Orai3 expression at both mRNA (6.42 ± 0.42-fold the control, Figure 7C-a, *p* < 0.001) and protein levels (2.58 ± 0.17-fold the control, Figure 7E-a, *p* < 0.001). Additionally, the expression of Nanog and SOX-2 was higher in CD133^+^ cells compared to parental A549 cells recording, respectively, 3 ± 0.30 and 2.12 ± 0.12 (Figure 7C-b,c, *p* < 0.001) at the transcript level and 1.62 ± 0.025 and 3.53 ± 0.18-fold the control at the protein level (Figure 7D,E-b,c, *p* < 0.001). Similar experiments were performed on H23 cells treated with CDDP 2.5 µM during 72 h (Figure S5A,B). CDDP increased CD133^+^ population as well as the expression of Nanog, SOX-2 and Orai3 (Figure S5B-b). In these cells, the expression of STIM1, STIM2, and Orai1 remained not affected by CDDP treatment (Figure S5B-a). Interestingly, the ectopic overexpression of Orai3 in both cell lines also enhanced CD133^+^ cell population (Figure S5C,D) in which SOX-2 and Nanog expression are increased (Figure S5G,H) without affecting the expression of STIM1, STIM2 or Orai1 (Figure S5E,F).

As CD44^+^ cells express the pluripotency genes Nanog and SOX-2 and as it was characterized as the major marker expressed by H23 cells [30], we also sorted CD44^+^ population. CDDP increased the CD44^+^ mean of fluorescence by flow cytometry with ratio of 2.1 and 1.4, respectively (Figure S6A) as well as Nanog, SOX2 and Orai3 expression without any effect on STIM1, STIM2 or Orai1 expression (Figure S6B-a,b). Moreover, the ectopic overexpression of Orai3 in H23 cells increased SOX-2 and Nanog expression (Figure S6C-b). Here also, we did not notice any significant effect on Orai1, STIM2 or STIM1 expression (Figure S6C-a) arguing for an Orai3-specific effect on stemness markers expression in both cells.

### 2.8. Orai3 Induced SOCE in Cisplatin-Induced CD133^+^ A549 Cells and Is Involved in Cell Apoptosis

**Figure 8:**
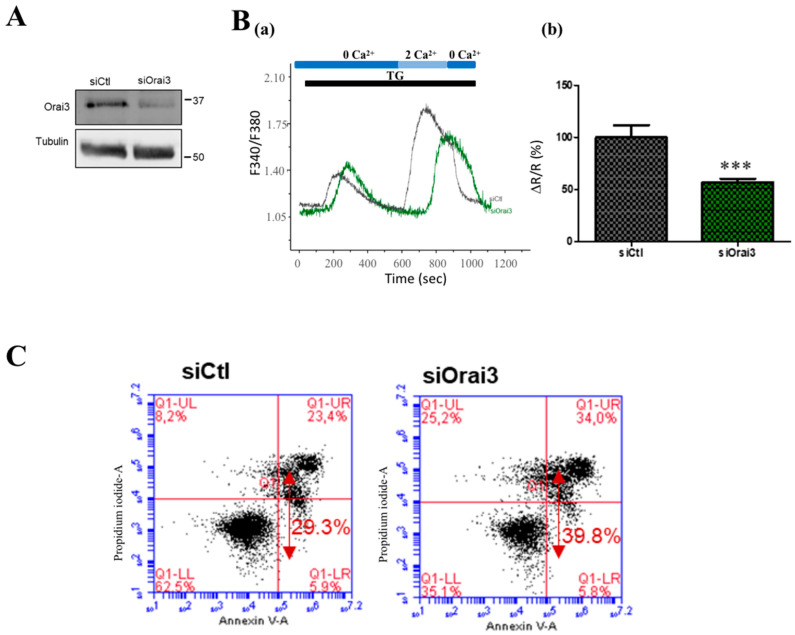

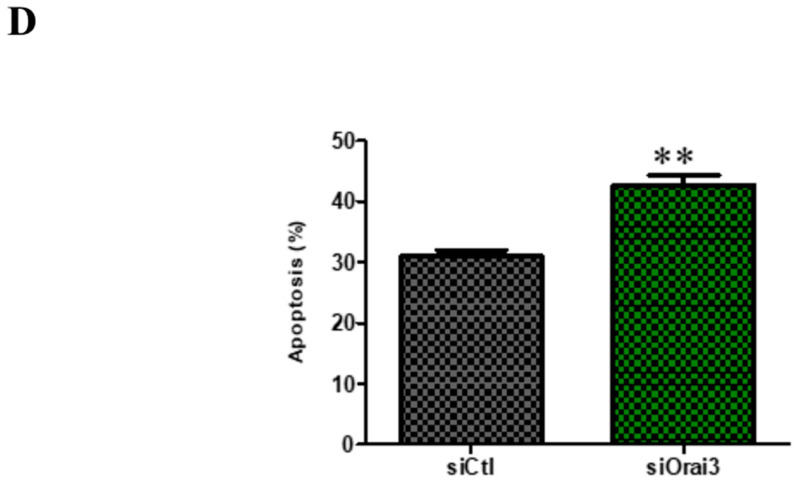
Function of Orai3 channel in CDDP-induced CD133^+^ A549 cells in calcium entry and apoptosis. Transfection validation: protein expression of Orai3 in CD133^+^ cells transfected with siCtl and siOrai3 (**A**). Traces representing the SOC entry measured with the ratio F340/F380 using calcium imaging technique (**B-a**). Cells were exposed to 1 µM TG in the absence of extracellular Ca^2+^ which depletes the intracellular Ca^2+^ stores. Extracellular calcium concentration was then brought to 2 mM in order to induce SOCE. Quantification of the SOCE by measuring the ratio of the peak amplitude over the initial (**B-b**). (siCtl: *n* = 60, siOrai3: *n* = 52, *N* = 3, *** *p* < 0.001, Student’s *t*-test, *n*: number of cells, N: number of passage). Flow cytometric analysis of early, late apoptosis and necrosis in CD133^+^ cells transfected with siCtl or siOrai3 (**C**). Bar chart represents the percentage of total apoptosis in each condition in CD133^+^ cells (**D**). Student’s *t*-test, *** *p* < 0.001 ** *p* < 0.01, *N* = 3.

After verifying the efficiency of siOrai3 transfection in CD133^+^ cells (Figure 8A), we then carried out calcium imaging experiments according to the SOCE protocol. While −37% reduction, in nonsorted CDDP-treated A549 cells, was observed upon siOrai3 (Figure S3C,D), siOrai3 led to a 43.37 ± 9.29% reduction of SOCE in CDDP-induced sorted CD133^+^ A549 cells (Figure 8B, *p* < 0.001). We used flow cytometry to assess the influence of Orai3 downregulation on apoptosis in CD133^+^ cells. Figure 8C,D showed that Orai3 silencing induced more cell apoptosis (42.73 ± 1.55%) compared to control cells transfected with siCtl, 72 h after transfection (31.03 ± 0.88%, *p* < 0.01).

### 2.9. Regulation of Orai3 and Stemness Markers Expression in CD133^+^ A549 Cells Induced by CDDP through PI3K/AKT Pathway

**Figure 9:**
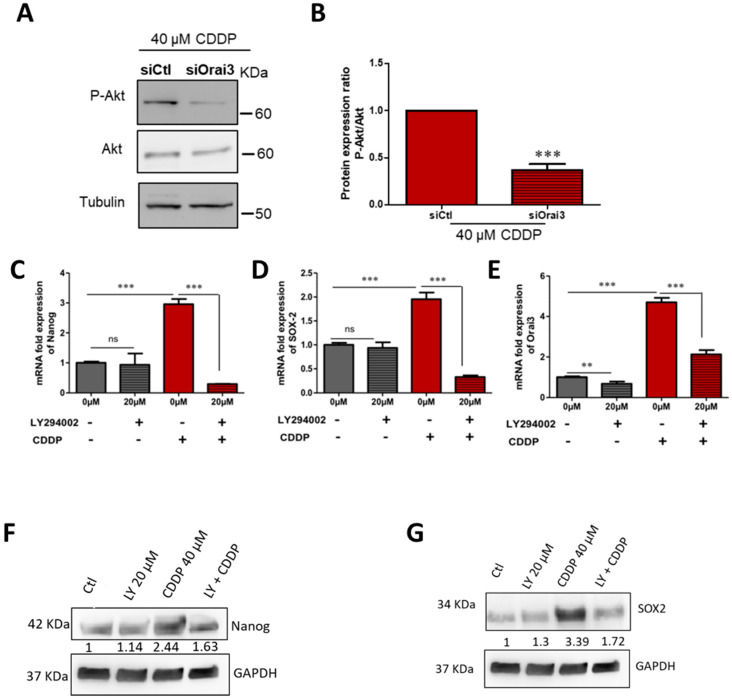
Regulation of Orai3 and stemness markers expression in CD133^+^ A549 cells induced by CDDP by PI3K pathway. Representative Western blotting of P-Akt and Akt proteins in A549 cells transfected with si-Orai3 or siCtl in the presence of CDDP (**A**) and respective protein expression (**B**). Data are presented as mean of three independent experiments ± SEM, Student’s *t*-test, *** *p* < 0.001. Relative mRNA expression with respect to GAPDH of Nanog (**C**), SOX-2 (**D**), and Orai3 (**E**) in CD133^+^ cells after 48 h CDDP treatment in the absence and presence of 20 μM LY294002 inhibitor. Relative protein expression of Nanog (**F**) and SOX-2 (**G**). Data are presented as mean of three independent experiments ± SEM, ANOVA followed by Holm–Sidak post-test ** *p* < 0.01 ***; *p* < 0.001, ns: not significant.

As Orai3 regulates the Ca^2+^-dependent signaling pathway PI3K/AKT in NSCLC [21], we investigated its possible involvement in the expression of CSCs markers induced by CDDP. For this, we used a pharmacological inhibitor of PI3K pathway LY294002. Figure 9A shows a significant decrease in AKT phosphorylation in cells downregulating Orai3 in the presence of CDDP. The level of AKT phosphorylation was quantified by the ratio *p*-AKT/AKT, and the results show a decrease in the ratio in siOrai3 condition (from 1-fold to 0.33 ± 0.02-fold, Figure 9B, *p* < 0.001) compared to siCtl treated with CDDP. We also found that Nanog and SOX-2 expression was drastically reduced in CD133^+^ cells treated with LY294002 (20 µM) in the presence of CDDP. Nanog mRNA expression decreased from 2.96 ± 0.17-fold the control to 0.299 ± 0.004-fold (Figure 9C, *p* < 0.001) and SOX-2 mRNA decreased from 1.95 ± 0.14-fold the control to 0.328 ± 0.033-fold, Figure 9D, *p* < 0.001), this decrease was also validated at the protein level (Figure 9F,G). We also observed that treatment with LY did not affect the expression of Orai1 or STIM1 (Figure S7A,B). In addition, the expression of Orai3 in CD133^+^ cells incubated with CDDP was also reduced from 4.7 ± 0.23-fold the control to 2.15 ± 0.21-fold (Figure 9E, *p* < 0.001).

## 3. Discussion

Chemoresistance is a serious problem since it usually puts forward the tumor cells’ ability to relapse and to gain pro-metastatic capacity allowing them to survive, migrate and grow new tumors at sites that are distant from the primary tumor [31]. Growing evidence supports the existence of CSCs in different types of tumors and considers them as the major reason behind chemoresistance. In our current study, we are the first to analyze the evolution of Orai3 expression in lung cancer tissues and cell lines during the course of chemotherapy, and to further investigate the relationship between Orai3 and CSCs.

We found out, for the first time, that Orai3 expression level varies drastically in lung adenocarcinoma tissues and cell line following a platinum-based chemotherapy. Immunostaining of tissues from patient cohorts with primary lung adenocarcinoma showed that platinum-based chemotherapy regimens increases the expression of Orai3, but not Orai1. Likewise, the in vitro, treatment with CDDP enhanced Orai3, but not Orai1 nor STIM1 or STIM2 expression in two lung adenocarcinoma cell lines: A549 and H23 cells. The A549 lung adenocarcinoma cell model has been extensively utilized in lung cancer research and drug development and served as testing ground for new drugs in vitro and in vivo [32]. H23 is another lung adenocarcinoma cell line, in which Orai3 has been reported as a major component of SOC independently of Orai1 [21]. These two cell lines showed a different sensitivity to Cisplatin. H23 cells are more sensitive to CDDP (IC_50_ = 2.5 µM) than A549 cells (IC_50_ = 40 µM). This tendency of Cisplatin effect that we observed in both cell lines was consistent with that found in the literature [33,34].

In both cell lines, CDDP increased Orai3 expression along with an increase of Calcium entry and stem cell markers. However, the activation mode of Orai3 seems to be different in these cell lines although they are both adenocarcinomas. In H23 cells, CDDP treatment increased SOCE amplitude, basal calcium concentration and ER Ca^2+^ that are strongly reduced in siOrai3 conditions. Furthermore, Orai3 knockdown was accompanied by strong inhibition of 2-APB-mediated SOCE potentiation in H23 cells, further indicating that CDDP favors SOCE through Orai3 channels in this cell line. In contrast, in A549 cells our results are in agreement with an existence of endogenous heteromeric channels formed by Orai1:Orai3. Indeed, A549 cells express more Orai1 and Orai3 than H23 cells. In basal culture conditions, we showed that Orai1 mediates SOCE in this cell line and that Orai3 seems to negatively regulate Orai1 since, upon its silencing, SOCE amplitude increased. Such effect upon silencing Orai3 on SOCE has already been reported in different cell types including prostate cancer cells [30] and enamel cells [35].

Orai3 is also involved in the regulation of the basal calcium concentration suggesting its activation in our medium culture as shown in H23 cells [21]. Indeed, the perfusion of 2-APB (50 μM) induced a slight increase of intracellular calcium concentration, in a Thapsigargin-independent manner, that is completely suppressed in cells lacking Orai3. Moreover, the 2APB response is increased by CDDP and was also suppressed in siOrai3 cells. However, we did not observe statistically significant differences of CDDP effect on basal concentration despite an increasing tendency. This result could be explained by the important cellular heterogeneity of this cell line. An interesting result is that under CDDP treatment, Orai3 becomes a positive regulator of SOCE. Indeed, the SOCE increase under CDDP is reduced upon silencing of Orai3. Recently, it has been shown that changes in the stoichiometry of Orai1 and Orai3 not only affect the pharmacological profile of the channel [36], but also to modulate the SOCE mediated by the different assemblies of Orai subunits in the hexameric channel [37]. One hypothesis could be that CDDP deviates the ratio Orai3:Orai1 to its favor and that shifts a population from Orai1:Orai3 heteromeric to Orai3 homomeric in CD133^+^ cells. Indeed, this shift of Orai3 from a negative regulator to a component in the SOCE in A549 cells after treatment with CDDP could be explained by the increase in the population of stem cells in which Orai3 constitutes a component of SOCE. Our results showing that both Orai3 silencing or its ectopic overexpression decreased and increased SOCE, respectively, are in favor with our hypothesis. However, as Orai3 regulates basal calcium entry and also that induced by CDDP, we cannot exclude the contribution of a store independent Ca^2+^ entry (SICE) through Orai3 as ARC (arachidonic acid-activated Ca^2+^ entry) or LRC (arachidonic acid metabolite Leukotriene C4-activated Ca^2+^ entry) [38,39,40,41] in CDDP-response in A549 cells.

We also noticed in both cell lines and in A549 cells in particular that Orai1 silencing decreased SOCE induced by CDDP more than siOrai3. However, although Orai1 appears to be involved in CDDP-induced SOCE, this channel is not involved in CDDP-induced overexpression of CSC markers and therefore in the resistance to CDDP treatment. We also found that Orai3 confers the cell survival in the presence of CDDP, and this process was found to be calcium dependent. Recent work in breast cancer cells showed that the overexpression of Orai3 increased SOCE and confers resistance to apoptosis through p53 protein expression modulation via the PI3K/Sgk-1 signaling pathway [23].

Extensive efforts in the field of CSCs have shown that CSCs can be isolated from a heterogeneous population of established carcinoma cell lines using special cell surface markers, such as CD133 which helps in the identification of this population. We detected an increase of the expression of the two stemness markers Nanog and SOX-2 during the in vitro treatment of H23 and A549 cells with CDDP which was a clue for the presence of CSCs. It is well established that exposure to CDDP fortifies CSCs subpopulation where CDDP-resistant cells express high levels of stemness markers including SOX-2 and Nanog [42]. In our study, we confirmed this phenomenon where CDDP treatment during 2–7 days progressively enhanced the expression of Nanog and SOX-2. In addition, using flow cytometry, we detected a higher percentage of CD133^+^ CSCs after CDDP incubation compared to control condition. We then found that the sorted CD133^+^ had higher expression of Nanog, SOX-2, and Orai3 than parental A549 and H23 cells. Moreover, the increase of the transcriptional expression of Orai3, Nanog and SOX2 in CD133^+^-A549- and H23 cells when compared to nonsorted cells, demonstrates the increase in the population of cancer stem cells. Consistent with our findings, Hsu and collaborators showed that CSCs isolated from primary tumors of NSCLC patients using CD133, expressed higher levels of Nanog and SOX-2 [10].

Interestingly, we found that the expression of Nanog and SOX-2 genes decreased upon Orai3 silencing suggesting a possible relationship between stemness acquisition and Orai3 expression. Rare published data has shown a correlation between CSCs and ion channels. One group suggested that the side population (SP) cells in NSCLC H460 cell line, which were less sensitive to gefitinib, have different mRNA expression patterns of the voltage gated potassium channels Kv channel subtypes compared to non-SP cells and that the combination of gefitinib and Kv blockers rendered the SP cells less resistant compared to only gefitinib treatment [43]. A second study proposed the usage of the VGCC α2δ1 subunit as a marker of gastric CSCs where α2δ1^+^ cells possessed CSCs properties. They also demonstrated that α2δ1 silencing sensitized the HGC-27 cells to CDDP [18]. The same subunit was also involved in CSCs properties of SCLC where α2δ1^+^ cells were able to form spheres in vivo and expressed high levels of stem cell-related transcription factors (including SOX-2 and Nanog) and drug resistance-related genes [19]. Another group proved the implication of VGCC of type L and T in ovarian CSCs where those channels were overexpressed in CSCs and their downregulation reduced the CSC like properties [17]. However, the implication of Orai channels in CSCs properties was only documented in one study where Orai1 was shown to be involved in enhancing CSCs where the overexpression of Orai1 in normal nontumorigenic oral epithelial cells stimulated the formation of cell populations with stem cell characteristics and showing self-renewal abilities [20].

Taking into consideration that cancer cell lines have been the most frequently used method to study lung CSCSs so far [44], we decided to investigate the Orai3 channel in sorted CD133^+^ cells. The Orai3 channel was found overexpressed and contributed to SOCE. Indeed, a significant decrease of SOCE was observed after Orai3 silencing in these cells suggesting that Orai3 tends to be a major participant in SOCE in this CD133^+^ subpopulation. This result could support the hypothesis that Orai3 mostly contributed in CSCs population after CDDP treatment. Moreover, the ectopic upregulation of Orai3 expression enhanced stemness markers expression: Nanog and SOX-2 and promoted apoptosis resistance to CDDP-treatment as silencing of Orai3 in CDD133^+^ decreased Nanog and SOX-2 expression as well as apoptosis rate.

We also investigated the signaling pathway by which Orai3 could confer resistance to CDDP. It has been reported that the activation of the PI3K/AKT cascade is a critical step of survival signaling which mediates apoptosis inhibition via multiple mechanisms [45,46]. The PI3K/AKT signaling is a core pathway for stemness acquisition and maintenance in cancer cells [47]. Furthermore, in lung cancer, the PI3K/AKT pathway was found to be central to cell survival and proliferation and was detected to be constitutively activated [48]. In addition, the Orai3 channel was reported to regulate AKT phosphorylation in NSCLC cells [21] which is consistent with our findings in A549 cells where Orai3 silencing was able to reduce AKT phosphorylation in CDDP-containing culture. Moreover, the expression of SOX-2 and Nanog was reduced after the inhibition of the PI3K/AKT pathway using a pharmacological inhibitor, suggesting a possible link between Orai3 expression/PI3K/AKT signaling and CSCs markers expression. Interestingly, PI3K/AKT seems to regulate the expression of Orai3 induced by CDDP.

## 4. Conclusions

In conclusion, both Orai1 and Orai3 are expressed in NSCLC cells. The increase of Orai3 expression in patient tissues after platinum-based treatment was correlated to bad prognosis. In vitro, CDDP was able to increase SOCE through the increase of Orai3 expression which in turn shifted the ratio of Orai1:Orai3 in favor of Orai3. Orai3, by regulating calcium entry, increased the expression of CSCs markers Nanog and SOX-2 probably via the PI3K/AKT pathway. Interestingly, PI3K/AKT seems to be involved in the upregulation of Orai3 expression induced by CDDP. Thus, Orai3 that has been identified as an independent prognostic marker of survival and metastasis in lung adenocarcinoma [22] can be also used as a future strategy in targeting CSCs subsets in CDDP resistant NSCLC tumors. However, further investigations are needed to understand the stoichiometry of Orai isoforms in CDDP treated A549 cells and also the molecular mechanisms that control this functional switch of Orai isoforms.

## 5. Materials and Methods

### 5.1. Ethics Statement

This study was conducted according to “Comité Consultatif de Protection des Personnes dans la Recherche Biomédicale de Picardie”. Surviving patients provided their written consent for their clinical information to be included in the study.

### 5.2. Characteristic Population of Prospective Cohort

Fifteen patients with advanced primary lung adenocarcinoma were subjected to biopsies between 2014 and 2015 at the University Hospital of Amiens and Hospital of Saint Quentin. The majority of patients were male (*n* = 9/15, 60%). The median age of patients at the time of diagnosis was 61.3 years (range: 42; 81). Among those with data, 86.7% of tobacco smokers (15/17) with a mean of 47 pack-years of cigarettes. The mean time between biopsy before chemotherapy and biopsy after chemotherapy was 57.6 days ± 6.5. Each patient was treated by a combination of carboplatin-pemetrexed, carboplatin-pemetrexed-bevacizumab, or carboplatin in combination with gemcitabine or taxotere. Biopsies were taken twice from each patient: Once before and another after chemotherapy, and immunohistochemistry was performed on the specimen using Orai3 and Orai1 antibodies. Slides were reviewed to confirm the diagnosis of lung adenocarcinoma based on histological and immunohistochemical profile. Clinical parameters at presentation (sex, age at diagnosis, smoking status, treatment by systemic chemotherapy, response evaluation criteria in solid tumors (RECIST 1.1)) were recorded for all cases.

### 5.3. Quantification of Staining Expression by Immunohistochemistry

The immunohistochemical analysis was performed as previously reported [22]. Briefly, formalin-fixed, paraffin-embedded 4 μm thick sections were first deparaffinized in xylene and rehydrated in ethanol. The endogenous peroxidase activity was blocked before antigen retrieval. Slides were then incubated with the primary antibody employing the automated immunostainer. The primary antibodies and their final dilutions were: Orai3 (rabbit polyclonal, 1:200, Sigma, Saint Louis, MO, USA) and Orai1 (rabbit polyclonal, 1:200, Sigma, Saint Louis, MO, USA). After that, the biotin-labeled secondary antibody was applied followed by the addition of avidin–biotin–peroxidase complex. Reactions were developed using DiAmino-3,3Benzidinetetrahy-drochloride (DAB) substrate solution (iVIEW DAB Detection Kit, Ventana, Meylan, France). The tissues were counterstained with hematoxylin. A negative control was established following the same procedure, but without the addition of the primary antibody. Two independent observers quantitatively evaluated, in double blind, the staining expression (×40 objective and ×10 eyepiece) of each specimen. To evaluate the relative IHC staining intensities in lung adenocarcinoma tissue samples, immunoreactivity was evaluated by H scoring system [49]. Briefly, stained adenocarcinoma cells were further classified into strong or weak positive cells, and the H scores were subsequently generated by adding together 3×% strongly stained cells, 2×% moderate stained cells, 1×% weakly stained cells, and 0×% negative cells. Accordingly, the staining score of Orai1 or Orai3 expressions is referred to as the Orai1 or Orai3 expression.

### 5.4. Cell Culture

A549 and H23 cells were cultured in a medium composed of 1× MEM (Minimum Essential Medium), 10% FBS (Fetal Bovine Serum), 0.22% sodium bicarbonate, 20 mM HEPES, 0.1 mM non-essential amino acids, and 2 mM glutamax (Gibco, Life Technologies, Cergy Pontoise, France). A549 CD133^+^ sorted cells were grown in DMEM/F12, 0% FBS medium supplemented with 20 mM EGF, 20 mM bFGF (tebu-bio, Atlanta, GA, USA). Cells were placed at 37 °C in a humidified atmosphere rich in CO_2_ (5%) and a fresh medium was added each 48 h.

### 5.5. Reagents

All the used chemicals were purchased from Sigma, unless otherwise stated. Cells were treated with the chemical agents including CDDP (40 μM), LY294002 (20 μM), Wortmanin (1 µM) dissolved in DMSO. Low Ca^2+^-medium was established by the addition of 1.5 mM of EGTA.

### 5.6. Transient Transfection

Transfection was done using the nucleofection technology (Amaxa Biosystems, Lonza, Aubergenville, France) according to the Amaxa Biosystems protocol. Then, 10^6^ cells were transfected with 5 µg of siRNA directed against Orai3 (5’ GGG UCA AGU UUG UGC CCA U 3’ Eurogentec, Angers, France), with scrambled siRNA as a control (siControl 5’-CUG GAC AUG GAC CAA GUG GAC UU-3’, universal negative control Eurogentec).

### 5.7. Flow Cytometry Analysis and Cell Sorting

After 48 h of vehicle or CDDP treatments, A549 cells were harvested, washed and stained with PE-conjugated mouse anti-human CD133 (clone AC133, Miltenyi Biotech, Paris, France) or PE-conjugated mouse control isotype (Miltenyi Biotech) in PBS plus 2 mM EDTA and 0.5% BSA on ice. In all conditions, doublet discrimination was performed to gate only single cells and nonviable cells exclusion was assessed using 7-amino-actinomycin D probe (7-AAD). CD133^+^ A549 subpopulations were sorted by FACS (Fluorescence-Activated Cell Sorting). Cell analysis and aseptic cell sorting was performed with a FACSAria II flow cytometer (Becton-Dickinson, Franklin Lakes, NJ, USA). FACS data for three independent experiments were analyzed using the FlowJo V10 software (TreeStar, Inc., Ashland, OR, USA).

### 5.8. Real-Time Quantitative PCR (qRT-PCR)

Total RNA extraction from cell lines was carried out and followed by real-time PCR as previously described [50]. The relative amount of Orai3, SOX-2 and Nanog mRNAs in both cell lines were normalized to the endogenous control (GAPDH) using the Pfaffl method [51].

### 5.9. Western Blot Analysis

RIPA buffer (containing protease and phosphatase inhibitor cocktail, Sigma Aldrich, Saint-Quentin, France) was used for protein extraction. Proteins from each condition were subjected to SDS-PAGE and then transferred to nitrocellulose membranes, which were blocked with 5% BSA and incubated afterwards with primary antibodies against anti-Orai3 (1:300, Sigma Prestige, Saint Quentin, France), anti-SOX-2 (1:500, Sigma Aldrich), anti-Nanog (1:500, Cell Signaling, Saint Quentin, France), anti-Akt (1:1000, Cell signaling), anti-*p*-Akt (Ser473) (1:400, Cell Signaling). Tubulin antibody (1:2000, Sigma Aldrich) or GAPDH (1:4000, abcam, Cambridge, UK) were used as an internal control. Secondary antibodies, coupled to horseradish peroxidase, were then utilized. Bound antibodies were visualized using ECL chemiluminescent substrate (GE Healthcare, Saclay, France) and quantified using the densitometric analysis option in the Bio-Rad image acquisition system (Bio-Rad Laboratories, Marnes-la-Coquette, France). (Original blots are included in File S1 in the Supplementary Materials). 

### 5.10. Cell Viability and Mortality

Cell mortality was assessed using Trypan Blue method. LC cells were grown in 35 mm Petri dishes at a density of 1 × 10^5^ cells, 24 h after seeding, cells were treated with different agents EGTA, CDDP or LY294002. Cell count was done six times using the standard Malassez cell method and the results were expressed as the percentage of dead cells normalized to control.

Cell viability estimation was done by the standard MTT test. Cells were usually seeded at a density of 6×10^4^ cell in 6-well plates. The duration of the different treatments was 48 h. MTT was diluted to 0.5 mg/mL in culture medium and cells were incubated at 37 °C during 45 min. The cells containing the formazan crystals were dissolved in DMSO and the absorbance was measured by spectrophotometer at 550 nm (Infinite P200 PRO™ TECAN) via Tecan-i-control software (1.8 SP1, Salzburg, Austria).

### 5.11. Apoptosis Analysis

To evaluate the percentage of apoptotic cells, we measured the exposure of phosphatidylserine residues at the outer plasma membrane by FITC Annexin V Apoptosis Detection Kit I (BD Biosciences Pharmingen, Le Pont-de-Claix, France). Detached and adherent cells were collected, washed twice in ice-cold PBS and resuspended in 1× binding buffer. Following the staining, the samples were analyzed with BD Accuri C6 flow cytometer.

### 5.12. Calcium Imaging

Cells, seeded on glass coverslips, were incubated with 3 μM Fura-2/AM (Sigma) for 45 min at 37 °C. Then, the cells were washed and kept in extracellular saline solution containing 145 mM NaCl, 5 mM KCl, 2 mM CaCl_2_, 1 mM MgCl_2_, 5 mM glucose, 10 mM HEPES (pH = 7.4). The coverslip was placed on a Zeiss microscope chamber equipped for fluorescence. Fura-2 fluorescence was excited at 340 and 380 nm using a monochromator (polychrome IV, TILL Photonics, Planegg, Germany), and captured by a Cool SNAPHQ camera (Princeton Instruments) after filtration through a long-pass filter (510 nm). Metafluor software (version 7.1.7.0, Molecular Devices, St. Grégoire, France) was used for signal acquisition during the experiment and analysis of the data. The [Ca^2+^]_i_ concentration was derived from the ratio of the fluorescence intensities for each of the excitations wavelengths (F_340_/F_380_). During acquisition, cells were continuously perfused with the saline solution. SOCE was triggered using Tg which leads to Ca^2+^ influx.

### 5.13. Statistical Analysis

Illustrated data are presented as mean ± SEM (standard error of mean), where N refers to the number of cell line passages and *n* to the number of cells. All experiments were carried out in triplicates, *N* = 3. The mean values of two groups were compared by the Student’s t-test or Mann–Whitney rank sum test, using, GraphPad Prism software. Mean values of more than two groups were tested using two-way analysis of variance (ANOVA) followed by Holm–Sidak post test or Dunnett post test (when all groups are compared to the control group). Differences between the values were considered significant when *p* < 0.05 where the *p*-values < 0.05, <0.01, and <0.001 are depicted as *, **, and ***, respectively.

## Data Availability

Data is contained within the article or supplementary material.

## Supplementary Materials

The following are available online at https://www.mdpi.com/article/10.3390/cancers13102314/s1, Figure S1: A dose–response curve for CDDP in H23 (A) and A549 (B) cells where the percentage cell survival is plotted against the logarithm of treatment concentrations to obtain the IC_50_ in each cell line, Figure S2: mRNA fold expression normalized to GAPDH in cells transfected with siCtl and siOrai3, Figure S3: Measurement of Calcium entry in A549 cells. Figure S4: Relative mRNA expression of Orai1, Orai3, STIM1, Nanog and SOX-2 in A549 cells 4 days after CDDP (5 µM) treatment (A). Figure S5: Flow cytometry representative histograms showing that 2.5 µM Cisplatin treatment increased H23-CD133 expression with respect to control and its respective quantification (A), Figure 6: Flow cytometry representative histograms showing that 2.5 µM Cisplatin treatment increased CD44 expression with respect to control in H23 cells and its respective quantification (A). Figure S7: Western blot representing Orai1 (A) and STIM1 (B) protein expression in A549 cells after CDDP treatment in the presence of 20 µM LY, File S1: Original western blot images.
