# Supplementary material for: Orai3-Mediates Cisplatin-Resistance in Non-Small Cell Lung Cancer Cells by Enriching Cancer Stem Cell Population through PI3K/AKT Pathway
Source: Cancers (Basel). 2021 May 12;13(10):2314. doi: 10.3390/cancers13102314 (PMC8150283; doi:10.3390/cancers13102314)
Supplement: Supplementary file 1 [file cancers-13-02314-s001.zip › cancers-1165867-supplementary.pdf]

cancers MDPI

Supplementary Material: Orai3-Mediates Cisplatin-Resistance in Non-Small Cell Lung Cancer Cells by Enriching Cancer Stem Cell Population through PI3K/AKT Pathway

Hiba Abou Daya, Hakim Ouled-Haddou, Sana Kouba, Nazim Benzerdjeb, Marie-Sophie Telliez, Charles Dayen, Henri Sevestre, Loïc Garçon, Frédéric Hague, Halima Ouadid-Ahidouch

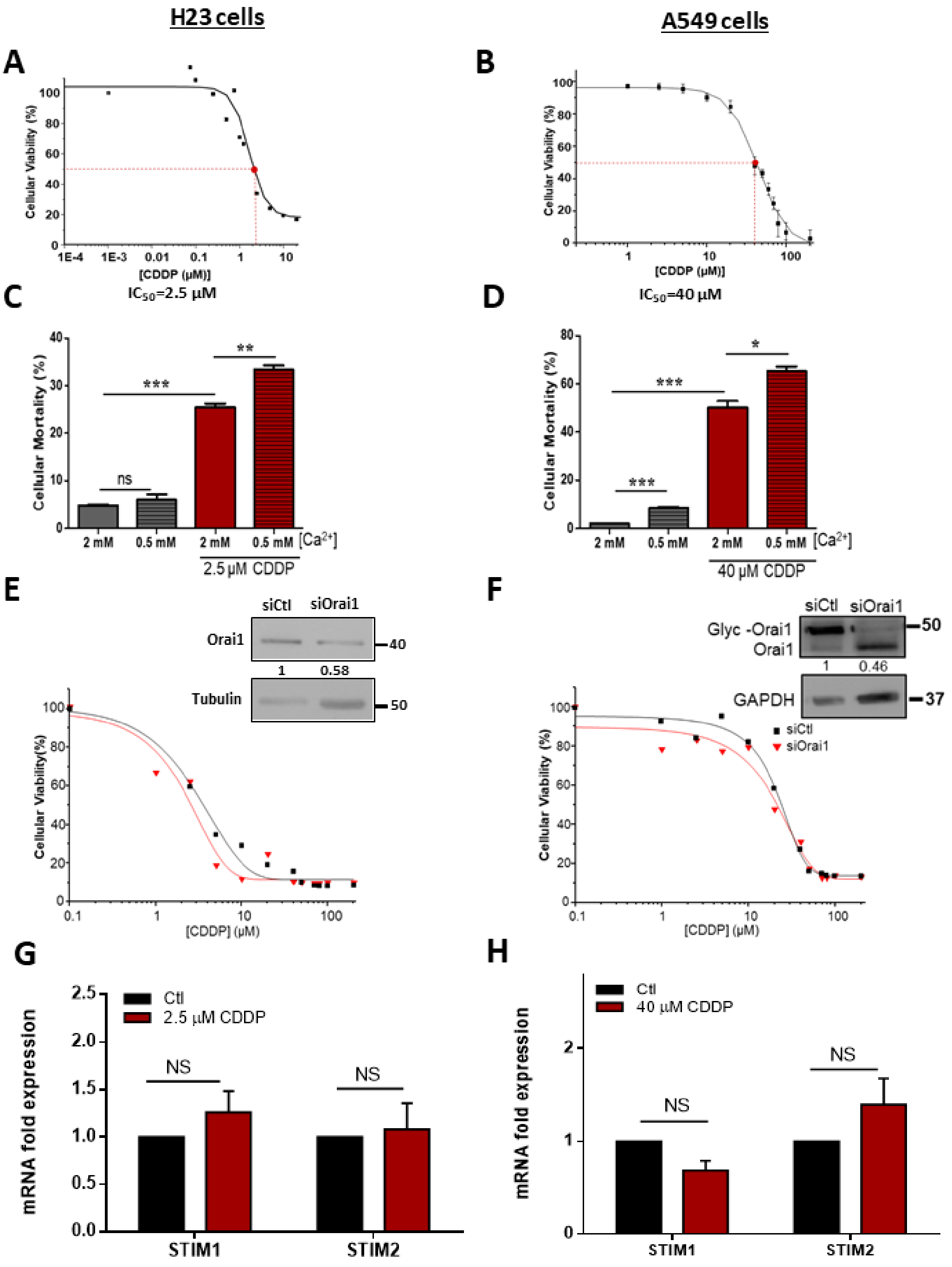

Figure S1. A dose–response curve for CDDP in H23 (**A**) and A549 (**B**) cells where the percentage cell survival is plotted against the logarithm of treatment concentrations to obtain the IC_{50} in each cell line. Data points are the means ± SD of duplicate determinations of triplicate measurements. Cellular mortality assessment using Trypan blue test in H23 (**C**) and

A549 (**D**) in normal calcium concentration (2 mM) and low calcium concentration (0.5 mM), $N = 3$. Values are presented as mean ± SEM. *** $p < 0.001$ ** $p < 0.01$ *$p < 0.05$ (ANOVA followed by Holm- Sidak posttest; ns: not significant). Percentage of cell survival is plotted against the logarithm of 48-hour treatment concentrations in control condition where cells were transfected with siCtl and the condition of siOrai1 in H23 (**E**), and A549 (**F**) transfected cells with their corresponding western blot revealing Orai1 expression after transfection with siCtl and siOrai1. mRNA fold expression of STIM1 and STIM2 normalized to GAPDH in H23 (**G**) and A549 cells (**H**) after 48-hour treatment with 2.5 μM and 40 μM CDDP respectively, values are presented as mean ± SEM, $N = 3$ (ANOVA followed by Holm-Sidak posttest; NS stands for: Not Significant).

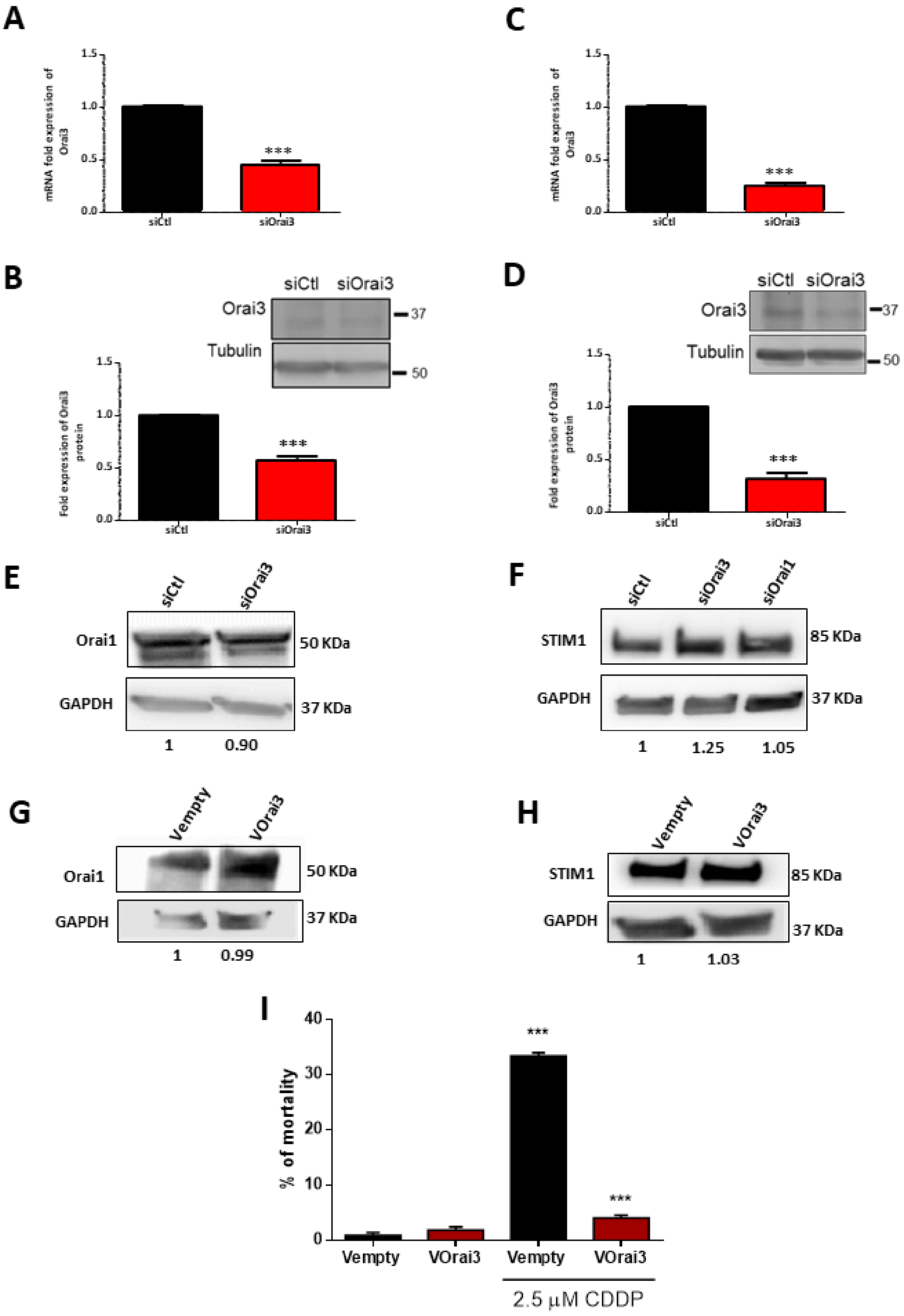

Figure S2. mRNA fold expression normalized to GAPDH in cells transfected with siCtl and siOrai3: H23 (**A**) and A549 (**C**) cells. Western blot representing Orai3 protein expression in H23 (**B**) and A549 (**D**) cells with their respective quantifications, $N = 3$ ± SEM. *** $p < 0.001$, Student's *t*-test. (**E**) and (**F**) Western blots representing Orai1 and STIM1 protein expression normalized to GAPDH with their respective quantification ratios after Orai3 silencing in A549 cells, $N = 3$. Orai3 knock

down did not have a significant effect on Orai1 or STIM1 protein expression. As to Orai1 silencing (**F**), STIM1 protein expression was not affected, *N*=3. (**G**) and (**H**) Western blots representing Orai1 and STIM1 protein expression normalized to GAPDH with their respective quantification ratios after Orai3 overexpression in A549 cells, *N* = 3. Ectopic overexpression of Orai3 did not affect the expression of Orai1 or STIM1 proteins. (**I**) Cellular mortality assessment using Trypan blue test in Orai3-overexpressing H23 control cells or treated with CDDP. Values are presented as mean ± SEM. *** *p* <0.001 *N* = 3 (ANOVA followed by Holm- Sidak posttest).

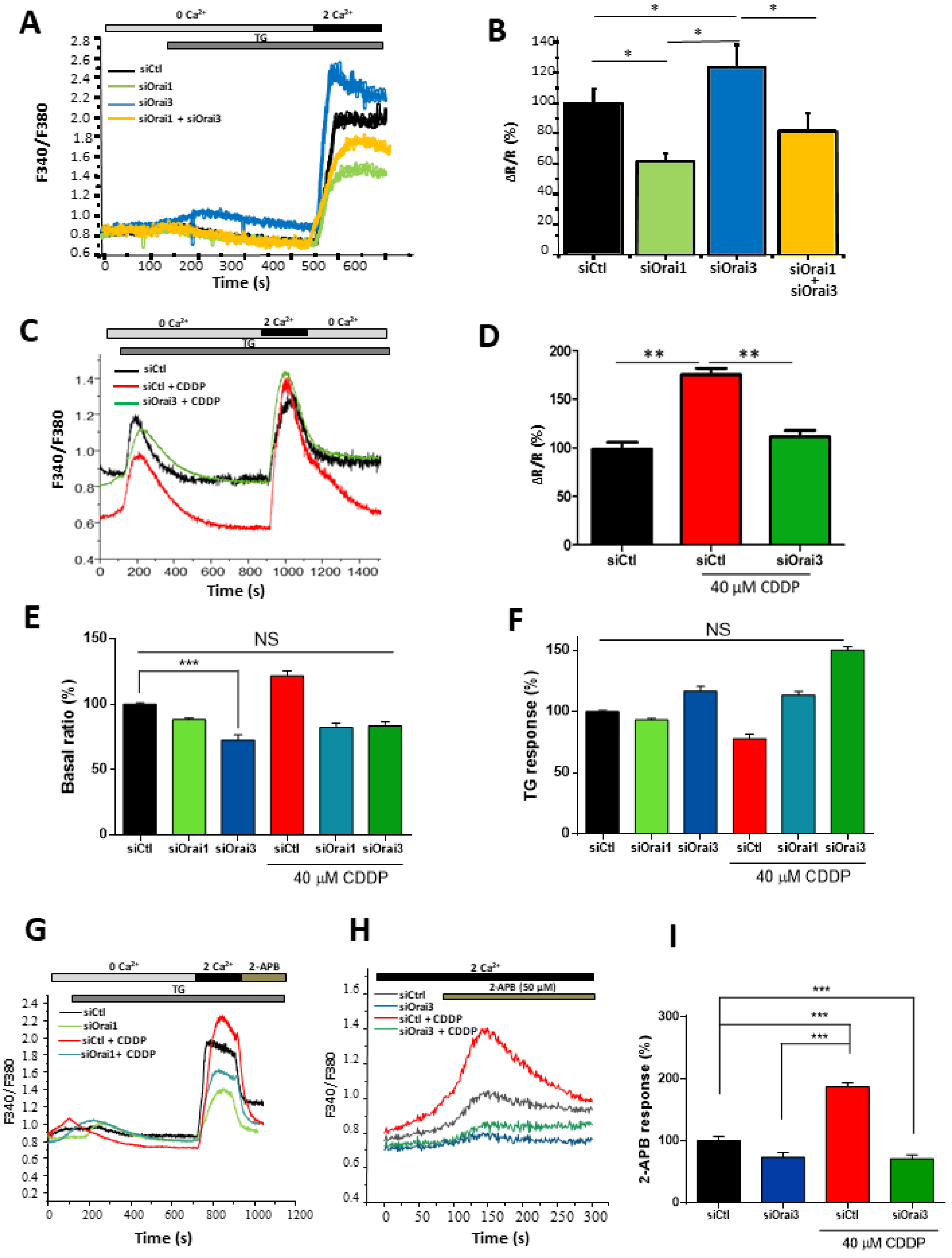

Figure S3. Measurement of Calcium entry in A549 cells. Traces representing the SOCE measured with Table 340. F380 using calcium imaging technique in A549 cells transfected with siOrai3 or siOrai1 (**A**) and upon the 48-hour treatment with CDDP (**C**). Cells were exposed to 1μM Tg in the absence of extracellular Ca^{2+} which depletes the intracellular Ca^{2+} stores. Extracellular calcium concentration was then brought to 2 mM in order to induce SOCE. Quantification of the SOCE by measuring the ratio of the peak amplitude over the initial values (**B**,**D**). Mean of basal Calcium ratio (**E**) as well TG-

response (**F**) in A549 cells transfected with siCtrl, siOrai1 or siOrai3 in the presence and absence of CDDP. (**G**) Traces representing the SOCE measured in A549 cells transfected with siOrai1 in the presence and absence of CDDP where 2-APB was perfused at the end of the protocol. (**H**) Effect of 2-ABP on basal Calcium entry in siCtl and siOrai3 conditions in the presence of CDDP with its respective quantification (**I**). All histograms are represented as the average ± SEM normalized to the control, of SOCE (**A–B** : siCtl: n = 200, siOrai1: n = 189, siOrai3: n = 190, siOrai1+ siOrai3: n= 120, N = 5), (**C–F**: siCtl: n = 175, siCtl + CDDP: n = 123, siOrai3 + CDDP: n= 113, N = 4), (**G**: siCtl: n = 90, siOrai1: n = 70, CDDP: n = 62, siOrai1 + CDDP: n = 54, N = 3), (**H–I**, siCtl: n = 80, siOrai3: n = 65, siCtl + CDDP: n = 45, siOrai3 + CDDP: n = 36, N = 3) $*p < 0.05$, $**p < 0.01$, $***p < 0.001$, NS: no significant, ANOVA followed by Holm- Sidak posttest;, n: number of cells, N: number of passage).

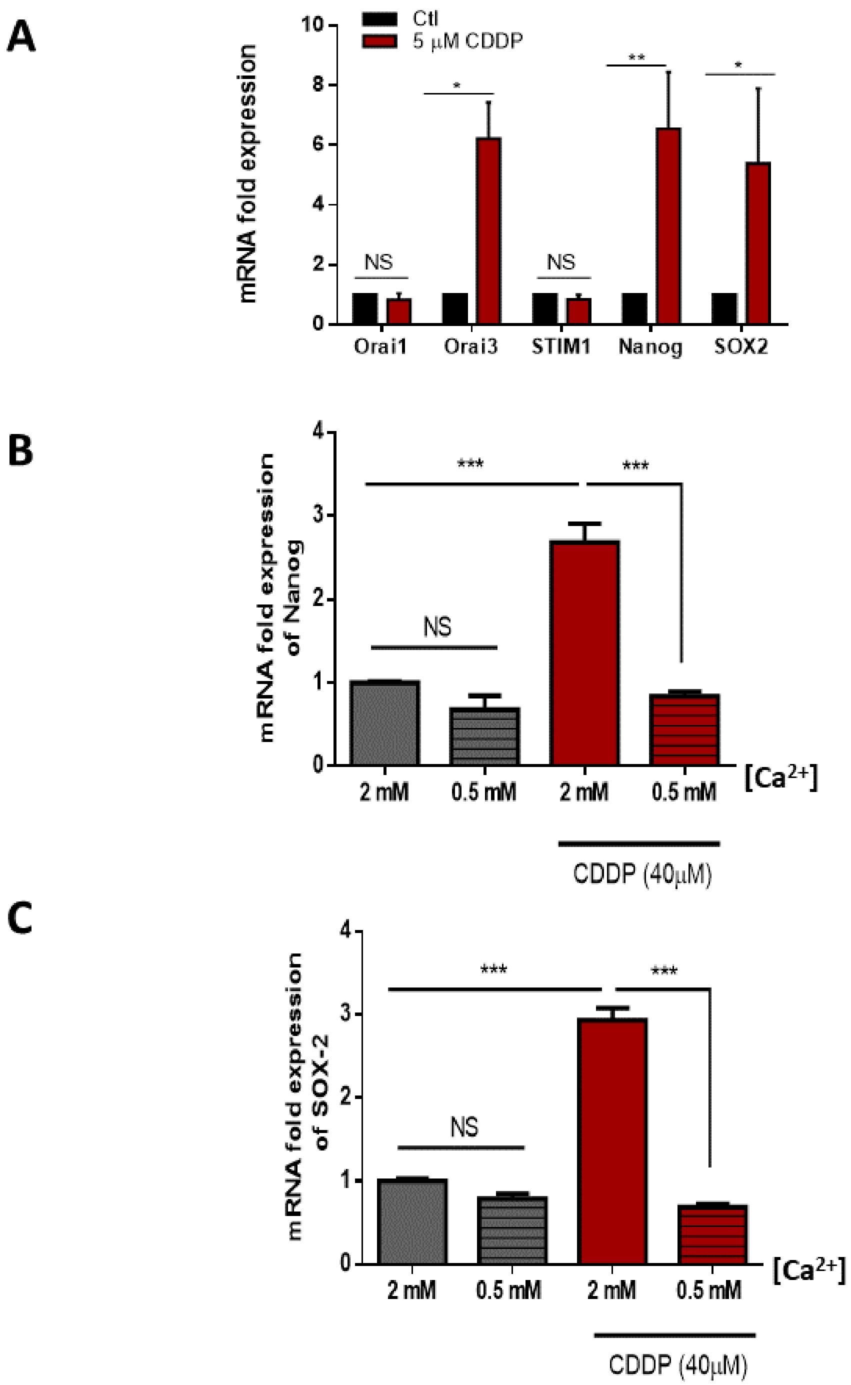

Figure S4. Relative mRNA expression of Orai1, Orai3, STIM1, Nanog and SOX-2 in A549 cells 4 days after CDDP (5 μM) treatment (**A**). The mRNA expression is normalized to GAPDH. mRNA expression of Orai3, Nanog and SOX-2 was significantly higher in treated cells with no significant effect on Orai1 or STIM1 expression, N = 3 (ANOVA followed by

Holm-Sidak test **p* <0.05; ***p* <0.01; NS stands for: not significant). Relative mRNA expression of Nanog (**B**) and SOX-2 (**C**) with respect to GAPDH after 48 h CDDP treatment in normal and low extracellular calcium concentrations, *N* = 5. (ANOVA followed by Holm-Sidak test **p* <0.05; ***p* <0.01; NS stands for: not significant).

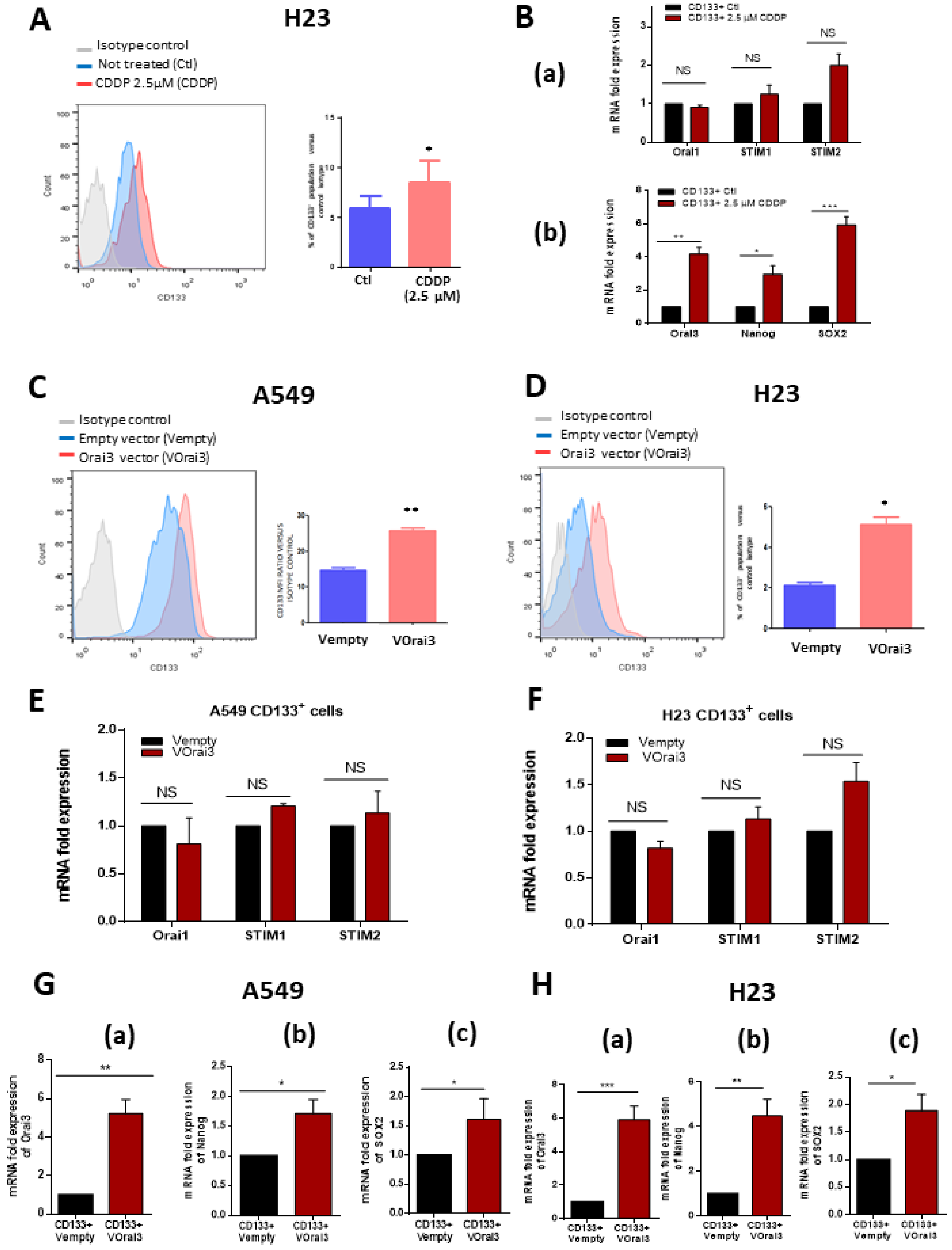

Figure S5. Flow cytometry representative histograms showing that 2.5 μM Cisplatin treatment increased H23-CD133 expression with respect to control and its respective quantification (**A**). Bars represent percentage values of CD133+ population of each condition relative to isotype *N* = 3. (**B-a**) Relative mRNA expression of Orai1, STIM1 and STIM2 in H23-CD133+ cells 4 days after CDDP (2.5 μM) treatment. The mRNA expression is normalized to GAPDH. No significant effect was

observed on Orai1, STIM1 or STIM2 expression, $N = 3$ (ANOVA followed by Holm-Sidak test; NS stands for: not significant). (**B-b**) Relative mRNA expression of Orai3, Nanog and SOX-2 in H23-CD133+ cells 4 days after CDDP (2.5 μM) treatment. The mRNA expression is normalized to GAPDH. Upon CDDP treatment, Orai3 as well as Nanog and SOX-2 transcriptional levels were significantly increased, $N = 3$ (ANOVA followed by Holm-Sidak test *p <0.05; **p <0.01; ***p < 0.001). Flow cytometry representative histograms showing that the ectopic overexpression of Orai3 in A549 (**C**) and H23 cells (**D**) induced a higher level of $CD133^+$ cell population with respect to control. Bars represent percentage values of $CD133^+$ population of each condition relative to isotype, $N = 3$. (**E**) and (**F**): Relative mRNA expression of Orai1, STIM1 and STIM2 in A549 and H23 Orai3-overexpressing $CD133^+$ cells, respectively. The experiments were performed 3 days after Orai3 plasmid transfection. GAPDH was used for normalization. Results indicate a non-significant effect on Orai1, STIM1 and STIM2 transcriptional expression, $N = 3$ (ANOVA followed by Holm-Sidak test; NS stands for: not significant). (**G-a**), (**G-b**) and (**G-c**): In A549 Orai3-overexpressing $CD133^+$ cells, Orai3 ectopic overexpression was followed by a significant increase of both Nanog and SOX-2 mRNA levels. (**H-a**), (**H-b**) and (**H-c**): The same expression profile was observed in H23 Orai3-overexpressing $CD133^+$ cells with an increase of Nanog and SOX-2 mRNA levels 3 days after Orai3 overexpression, $N = 3$ (Wilcoxon test *p <0.05; **p <0.01; ***p < 0.001).

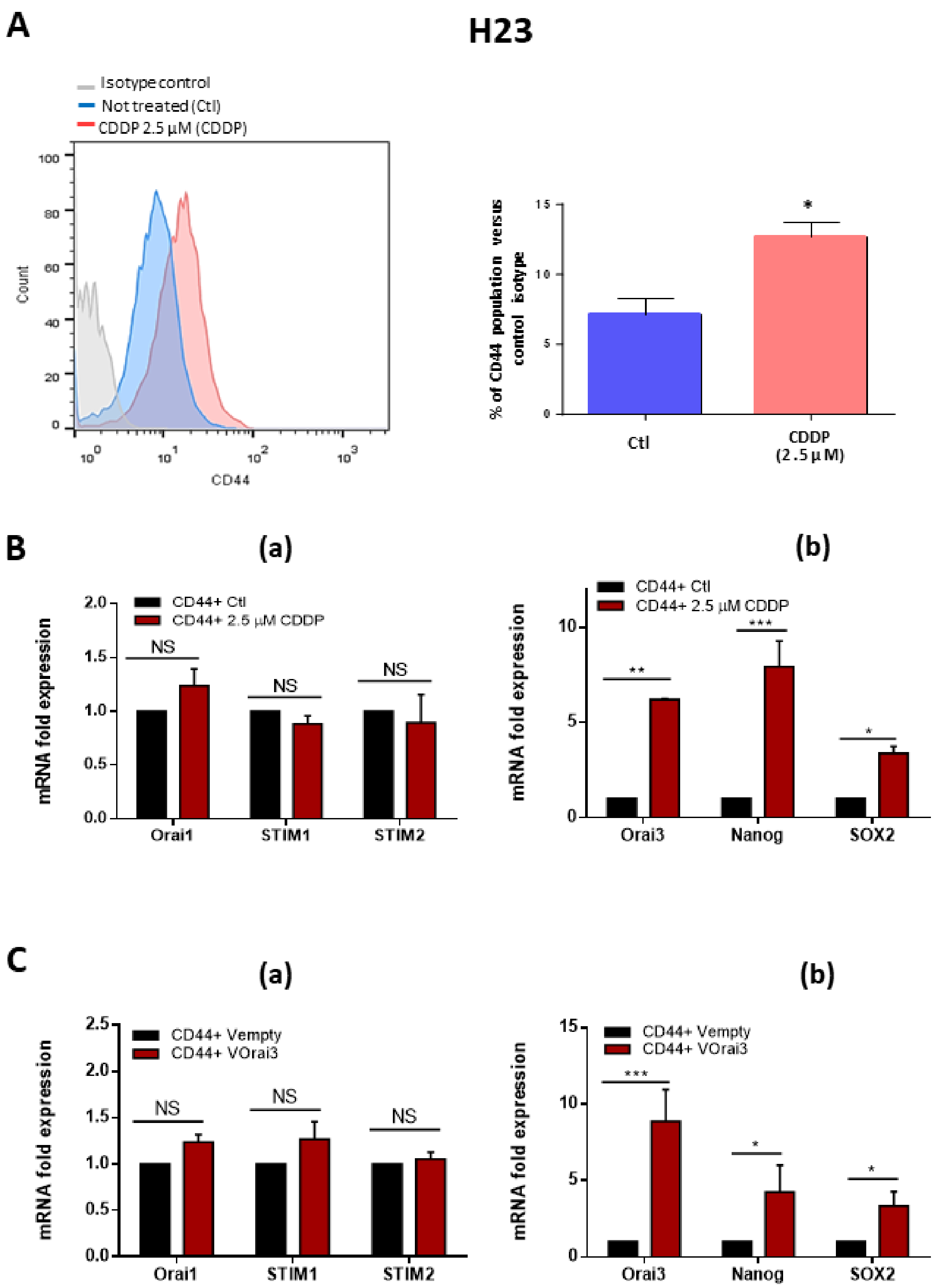

Figure S6. Flow cytometry representative histograms showing that 2.5 µM Cisplatin treatment increased CD44 expression with respect to control in H23 cells and its respective quantification **(A)**, *N* = 3. Bars represent percentage values of CD44⁺ population of each condition relative to isotype. (**B-a**): Relative mRNA expression of Orai1, STIM1 and STIM2 in H23-CD44⁺ cells 4 days after CDDP (2.5 µM) treatment. GAPDH was used for normalization *N* = 3. Results indicate a non-significant effect on Orai1, STIM1 and STIM2 mRNA levels. However, consequent to CDDP treatment, the mRNA levels of Orai3, Nanog and SOX-2 were significantly elevated (**B-b**) *N* = 3 (ANOVA followed by Holm-Sidak test *p <0.05; **p <0.01; ***p < 0.001; NS stands for: not significant). (**C-a**): In H23 Orai3-overexpressing CD44⁺ cells, Orai3 ectopic overexpression was followed by a significant increase of both Nanog and SOX-2 mRNA levels but no significant effect was observed on Orai1 or STIM1 mRNA levels (**C-b**), *N* = 3 (ANOVA followed by Holm-Sidak test; NS stands for: not significant).

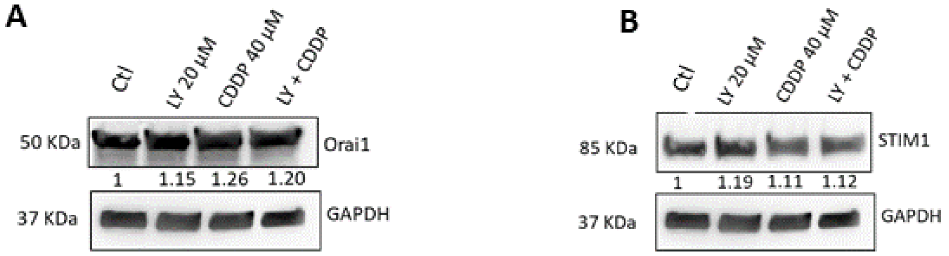

Figure S7. Western blot representing Orai1 (**A**) and STIM1 (**B**) protein expression in A549 cells after CDDP treatment in the presence of 20 μM LY. Data are quantified with respect to GAPDH, *N* = 3.
