# Supplementary material for: Orai3-Mediates Cisplatin-Resistance in Non-Small Cell Lung Cancer Cells by Enriching Cancer Stem Cell Population through PI3K/AKT Pathway
Source: Cancers (Basel). 2021 May 12;13(10):2314. doi: 10.3390/cancers13102314 (PMC8150283; doi:10.3390/cancers13102314)

Figure 2 A

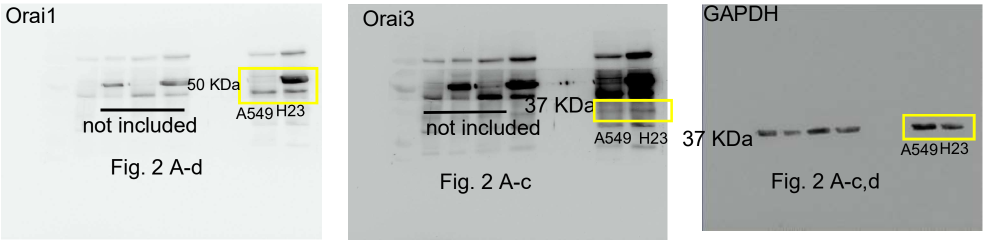

Figure 3 A,B and Figure 5 A

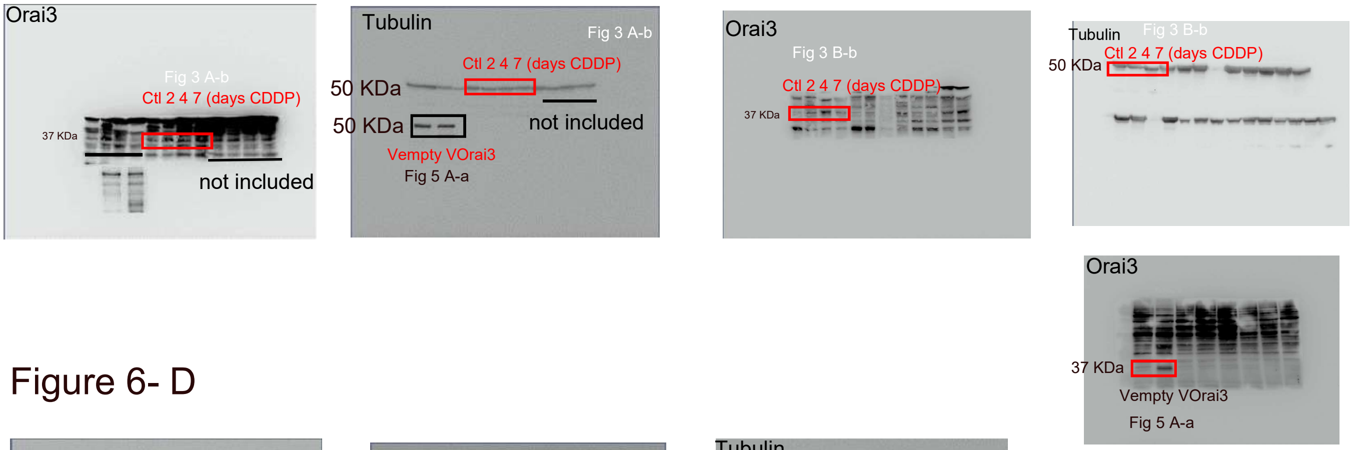

Figure 6- D

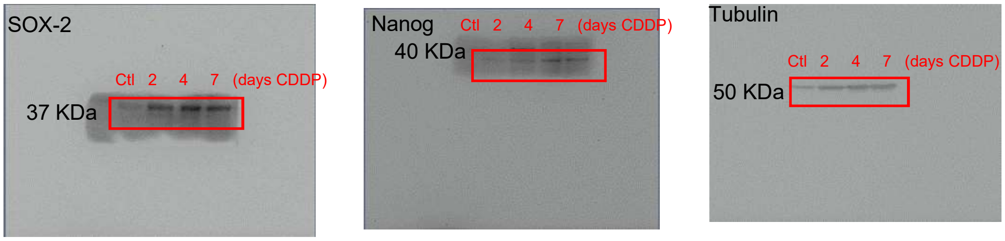

Figure 6- I

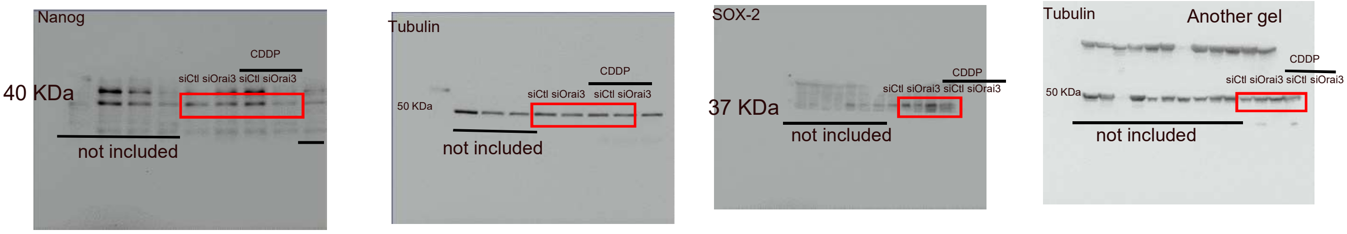

Figure 7- D

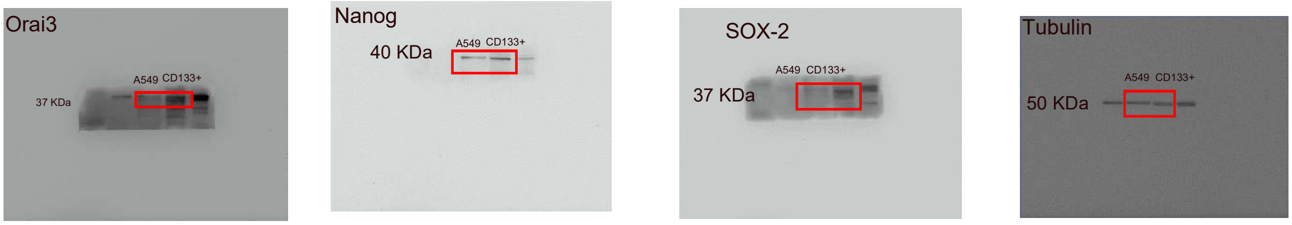

Figure 8-A

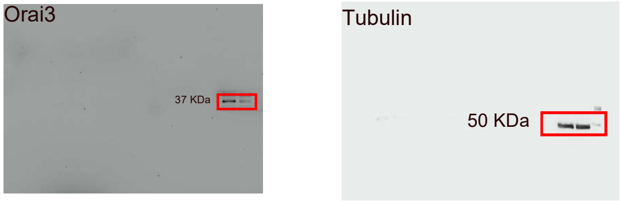

Figure 9-A

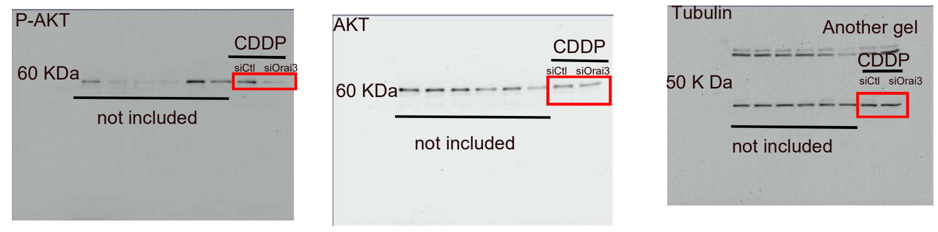

Figure 9-F

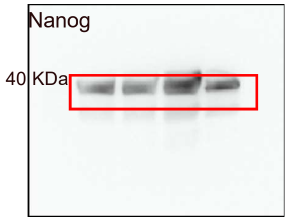

Figure 9-G

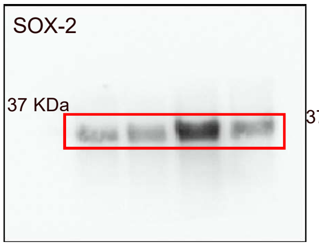

Figure 9-F,G

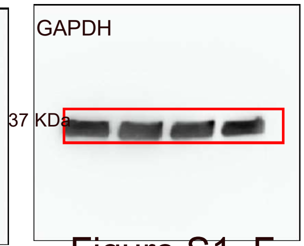

Figure S1, E

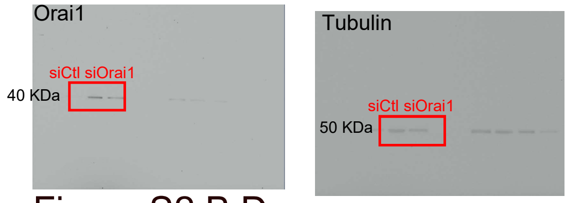

Figure S1, F

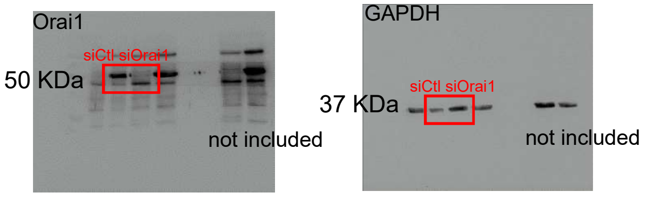

Figure S2 B,D

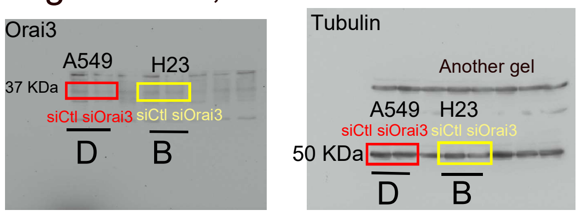

Figure S2 E

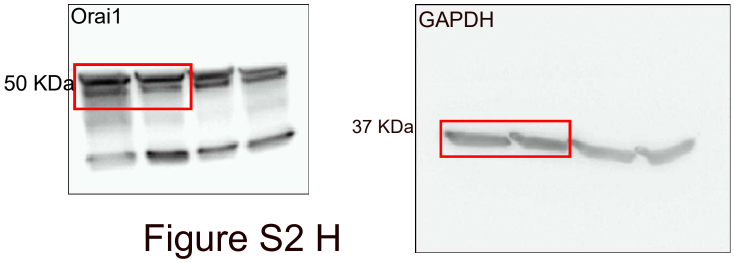

Figure S2 F

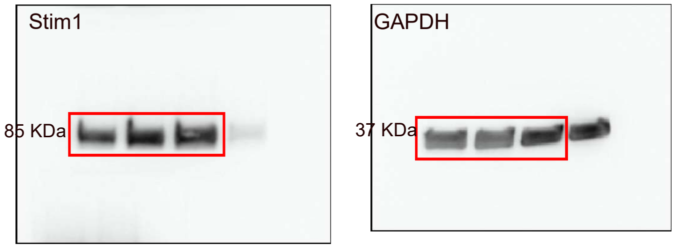

Figure S2 H

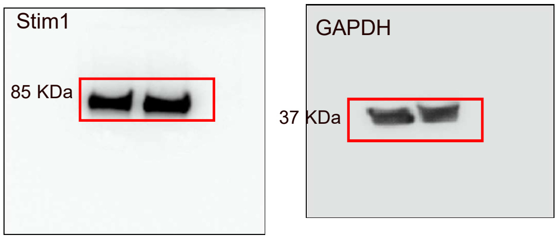

Figure S2- G

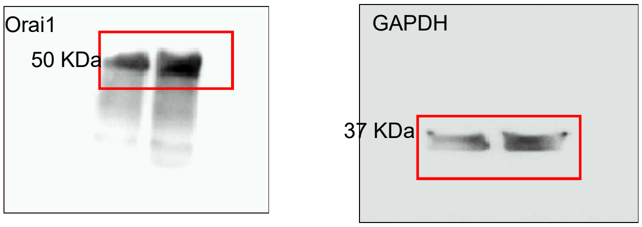

Figure S7 A,B

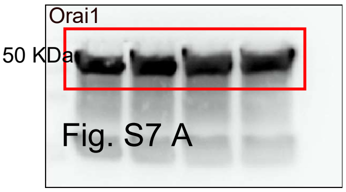

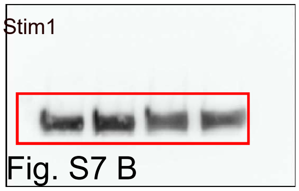

Figure S7 A,B

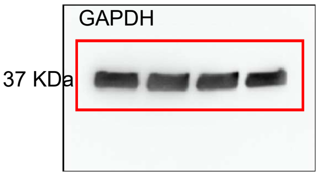

Supplement: Supplementary file 1 [file cancers-13-02314-s001.zip › File S1. Original western blot images.pdf]
